# Complex sexually dimorphic traits shape the parallel evolution of a novel reproductive strategy in Sulawesi ricefishes (Adrianichthyidae)

**DOI:** 10.1186/s12862-021-01791-z

**Published:** 2021-04-20

**Authors:** Tobias Spanke, Leon Hilgers, Benjamin Wipfler, Jana M. Flury, Arne W. Nolte, Ilham V. Utama, Bernhard Misof, Fabian Herder, Julia Schwarzer

**Affiliations:** 1 Zoologisches Forschungsmuseum Alexander Koenig, Adenauerallee 160, 53113 Bonn, Germany; 2 Carl Von Ossietzky Universität Oldenburg, AG Ökologische Genomik, Carl von Ossietzky-Str. 9-11, 26111 Oldenburg, Germany; 3 Ichthyology Laboratory, Indonesian Institute of Sciences (LIPI), JL. Raya Jakarta-Bogor Km. 46, Cibinong, 16911 Indonesia

**Keywords:** Maternal care, Ribs, Rib length, Sexual dimorphism, Sexual antagonistic selection, *Oryzias*, *Adrianichthys*

## Abstract

**Background:**

Pelvic brooding is a form of uni-parental care, and likely evolved in parallel in two lineages of Sulawesi ricefishes. Contrary to all other ricefishes, females of pelvic brooding species do not deposit eggs at a substrate (transfer brooding), but carry them until the fry hatches. We assume that modifications reducing the costs of egg carrying are beneficial for pelvic brooding females, but likely disadvantageous in conspecific males, which might be resolved by the evolution of sexual dimorphism via sexual antagonistic selection. Thus we hypothesize that the evolution of pelvic brooding gave rise to female-specific skeletal adaptations that are shared by both pelvic brooding lineages, but are absent in conspecific males and transfer brooding species. To tackle this, we combine 3D-imaging and morphometrics to analyze skeletal adaptations to pelvic brooding.

**Results:**

The morphology of skeletal traits correlated with sex and brooding strategy across seven ricefish species. Pelvic brooding females have short ribs caudal of the pelvic girdle forming a ventral concavity and clearly elongated and thickened pelvic fins compared to both sexes of transfer brooding species. The ventral concavity limits the body cavity volume in female pelvic brooders. Thus body volumes are smaller compared to males in pelvic brooding species, a pattern sharply contrasted by transfer brooding species.

**Conclusions:**

We showed in a comparative framework that highly similar, sexually dimorphic traits evolved in parallel in both lineages of pelvic brooding ricefish species. Key traits, present in all pelvic brooding females, were absent or much less pronounced in conspecific males and both sexes of transfer brooding species, indicating that they are non-beneficial or even maladaptive for ricefishes not providing extended care. We assume that the combination of ventral concavity and robust, elongated fins reduces drag of brooding females and provides protection and stability to the egg cluster. Thus ricefishes are one of the rare examples where environmental factors rather than sexual selection shaped the evolution of sexually dimorphic skeletal adaptations.

**Supplementary Information:**

The online version contains supplementary material available at 10.1186/s12862-021-01791-z.

## Background

Examples of repeated parallel or convergent evolution are omnipresent, i.e. the evolution of similar phenotypes shaped by the same selective regimes that may or may not trace back to a common ancestor [1–3]. Prominent examples in the animal kingdom are flight, advanced eyes [4], or the evolution of parental care, from the most common state of no-care to extended care [5], like placental viviparity [6]. In species that conduct care, selective burden is often unevenly distributed among the sexes and impacts sex specific life histories. In organisms with uni-parental care, costs of care only arise for one parent so selection to increase individual fitness can differ extremely between the sexes [7–9]. The resulting sexual conflict can be resolved by sex specific adaptations to parental care [10]. Nonetheless, only a few cases of sexually dimorphic traits are known that are not primarily shaped by sexual selection, but instead evolved as a trade-off between ecological constraints and adaptation to uni-parental care [11–15]. External bearing [16, 17] or egg brooding [18] is a form of parental care in which eggs are carried externally, attached to the parents body, sometimes by specialized structures, or in the parents mouth. In some of these cases, eggs are nurtured [19] by either of the parents. Compared with egg attendance, where parents remain with the eggs at a fixed location, egg brooding is particularly advantageous in variable environments [18], as brooding parents can move freely to escape egg-predation and unfavorable habitat conditions. Carrying the brood may however require anatomical adaptations, which can include the evolution of highly specialized tissues and organs, like brood pouches and incubating areas in seahorses and pipefishes [20, 21].

**Fig. 1:**
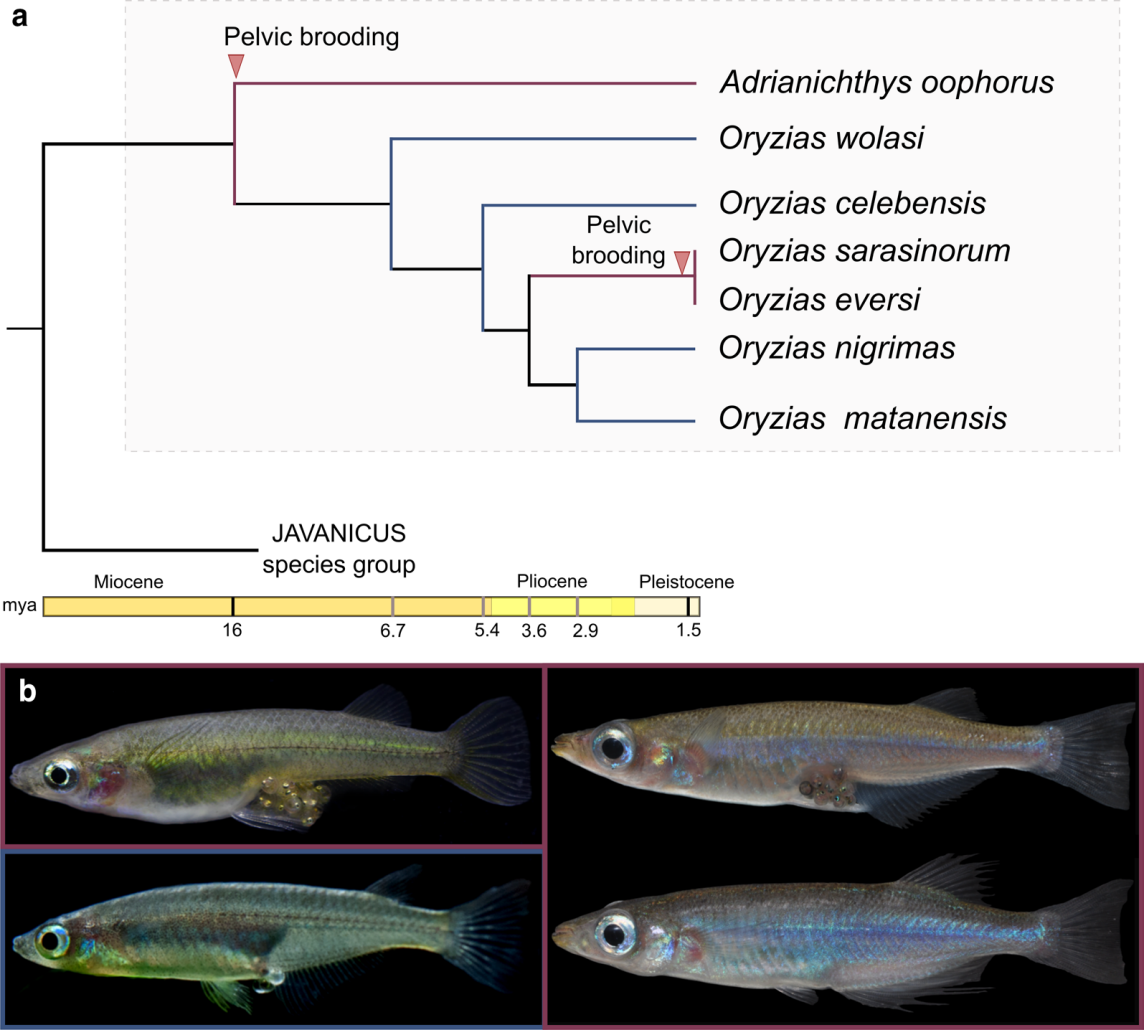
Study system: the endemic ricefishes of Sulawesi. **a** Simplified ricefish phylogeny with emphasis on Sulawesi ricefishes. Divergence times and phylogenetic relationships are based on [22]. Red arrows mark the assumed maximum ages for the origin of pelvic brooding in the two respective lineages [22]. **b** Left panel: Lateral view of females *Oryzias eversi* (top) and *Oryzias nigrimas* (bottom). The pelvic brooding *Oryzias eversi* carries a seven days old cluster of eggs. Eyes and pigments of the developing embryos are visible. Eggs of the transfer brooding species *Oryzias nigrimas* are about two hours old and will be deposited within the next few hours. The pelvic fins of female *O. nigrimas* are visibly shorter compared to *O. eversi*. Right panel: Brooding female (top) and male (bottom) of *Adrianichthys oophorus*

In Sulawesi ricefishes (Beloniformes, Adrianichthyidae) a specific form of external bearing called ‘pelvic brooding’ evolved from ancestral transfer brooding between 16 and 3 mya in large lacustrine *Adrianichthys* species [22] and about 1–1.5 mya within one lineage of closely related *Oryzias* species [22–24]. Most ricefishes are transfer brooders, which means that females carry a clutch of fertilized eggs via attaching filaments that protrude from their urogenital pore. The eggs are deposited a few hours after mating [25, 26]. In contrast, females of pelvic brooding species carry their eggs until the fry hatches; for up to 18 days after spawning [27]. Female pelvic brooders situate the egg cluster in a ventral concavity, while using their conspicuously elongated pelvic fins to cover the eggs (Fig. 1) [24, 28, 29]. Additionally, female pelvic brooders slow down oocyte maturation [27] and form a tissue that anchors the eggs’ attaching filaments inside the ovarian cavity [29]. This illustrates that the evolution of pelvic brooding from transfer brooding likely entails a set of physiological and anatomical adaptations. Details on most of these aspects, however, remain scarce and current knowledge on both transfer brooding and pelvic brooding is based mainly on single species studies (e.g. [24, 25, 27, 30]). Although ecological data on this exceptional reproductive strategy is limited, pelvic brooding was suggested to have evolved as adaptation to the absence of suitable spawning substrates in pelagic habitats [24]. Some morphological adaptations to pelvic brooding, namely elongated pelvic fins (first described by [31] and [32]) and the presence of a ventral concavity in females [28], are well known (see also [23, 24, 27, 29]). As few studies have performed comparative analyses among *Oryzias* species, data remained mostly descriptive, lacking information on sexual dimorphism (e.g. in *A. oophorus*) and have no statistical support (but see [24]). In most transfer brooding ricefish species, sex-specific differences in pelvic fin length were not described. Recent studies, however, reported sex-specific differences in pelvic fin length in the newly described transfer brooding species *Oryzias soerotoi* and *O. dopingdopingensis* [33, 34], indicating the need for more detailed analyses.

In the present study, we build upon prior knowledge and place it in a comparative framework of seven species, representing all phylogenetic lineages within the endemic ricefishes from Sulawesi ([22], Fig. 1). To gain a more conclusive picture regarding sex specific differences and skeletal adaptations to pelvic brooding, we used high-resolution µ-computed tomography (µ-CT) imaging and morphometrics and focus on adaptations of the ribs and pelvic fins. We further investigated, how changes in body shape in pelvic brooding females, i.e. the presence of the ventral concavity, affected the volume of their body cavity. We hypothesized that pelvic brooding females have a reduced body cavity volume, when compared to conspecific males or transfer brooding species. Since pelvic brooding is a form of uni-parental care, only the care-giver bears potential costs of egg carrying. Such costs can be higher energetic demands [35, 36] or an increased predation risk of the care-giver [26]. We thus assume, that modifications reducing the costs of egg carrying are beneficial for pelvic brooding females, but disadvantageous in conspecific males. The resulting sexual conflict could be resolved by the evolution of sexual dimorphism via sexual antagonistic selection. Hence, we hypothesized that the evolution of pelvic brooding gave rise to female-specific skeletal adaptations that are shared between both pelvic brooding lineages, but are absent in conspecific males and transfer brooding species.

## Results

### Specific ribs are shorter in females of pelvic brooding ricefishes

**Fig. 2:**
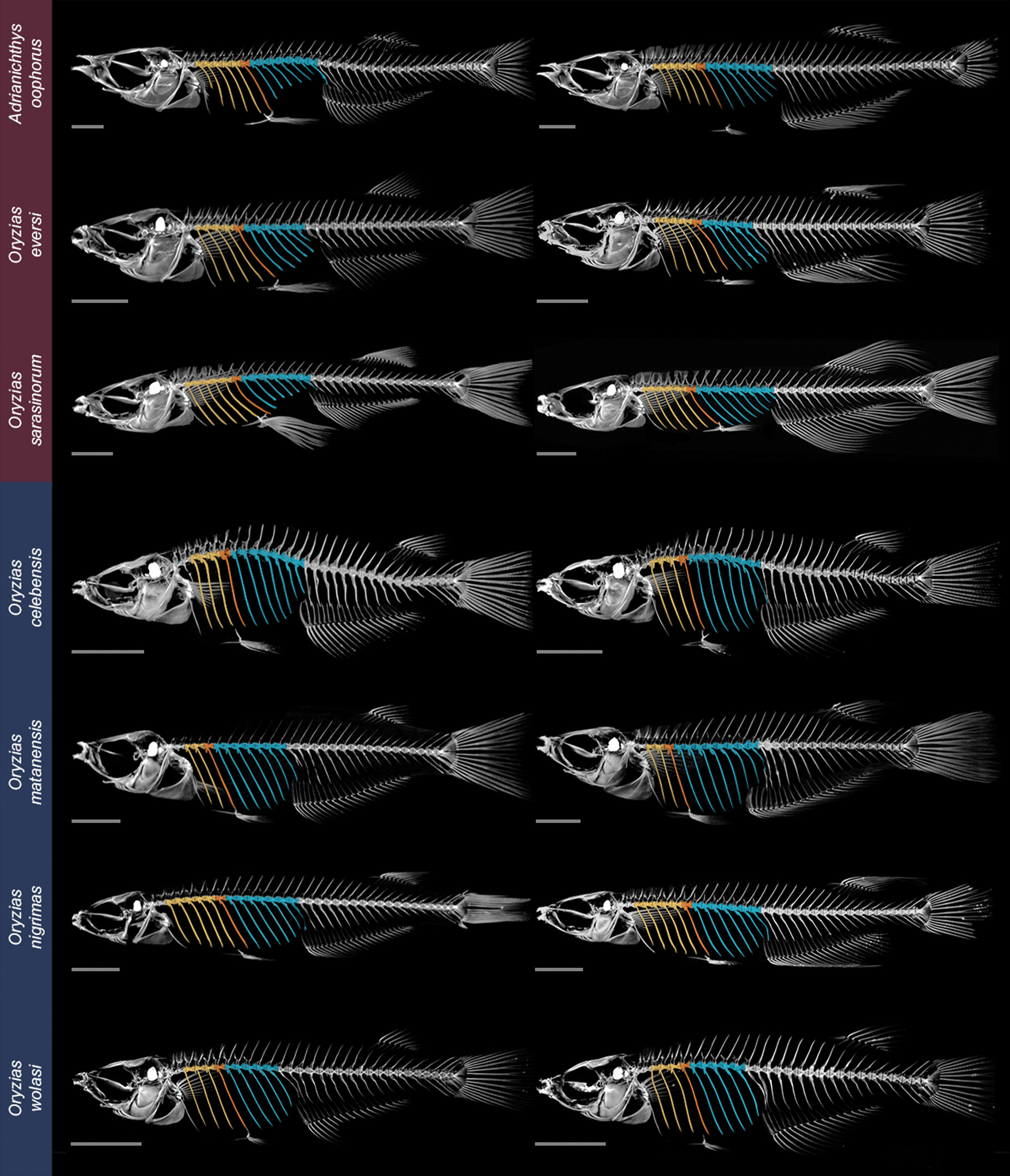
Maximum intensity projections of female (left) and male (right) ricefish species. Coloration of ribs indicates the rib above the insertion site of the pelvic fins in the pelvic girdle (R0, orange), as well as ribs anterior (yellow) and posterior (blue) to this position. Ribs are shorter into the caudal direction after the insertion of the pelvic fins at the pelvic girdle (blue colored ribs) in the three pelvic brooding females. Species names shaded in color on the left indicate pelvic brooding (dark red) and transfer brooding (dark blue) species, respectively

**Fig. 3:**
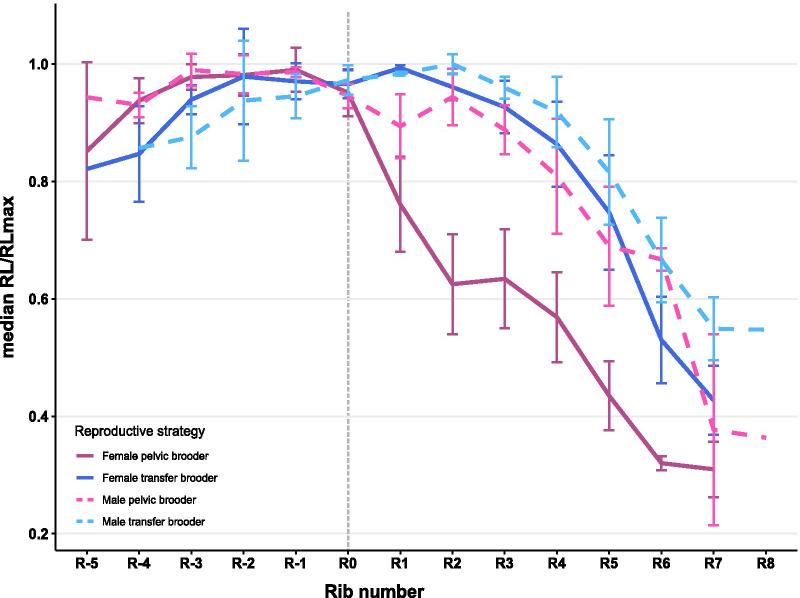
Relative rib length of female and male ricefishes in pelvic brooders and transfer brooders. The three pelvic brooding species and the four transfer brooding species were pooled, respectively; shown are the median relative rib length and the standard deviation. Relative rib length is calculated by dividing the length of each rib by the longest rib of the respective specimen. The grey line (R0) marks the position of the lateral process and the insertion of the pelvic fin rays at the pelvic girdle. Additional file 2: Fig. S1 shows data points per species

Intersexual differences in rib length were present in pelvic brooding ricefish species (*O. eversi*, *O. sarasinorum* and *A. oophorus*) and interspecific differences were present between females of pelvic brooding and transfer brooding (*O. celebensis, O. nigrimas, O. wolasi, O. matanensis*) ricefishes (Figs. 2, 3).

Across all ribs, standard length, i.e. the distance from tip of the snout to the caudal peduncle, was the predictor that was most often significant in linear mixed-effect models (LMMs) for rib length, rib height and rib gap (Additional file 1: Tables S1, S2, S3). A significant negative correlation of rib length and the interaction of sex and reproductive strategy was found for rib R0, located at the pelvic girdle (Fig. 5a), with males of pelvic brooding species having shorter ribs than conspecific females (LMM: p = 0.0128, F_1,19.8_ = 7.4883, β = − 0.74). For the three ribs located posterior of the pelvic girdle (R1, R2 and R3), we found a significant correlation of rib length with reproductive strategy (LMM: R1 p = 0.0477, F_1,8.4_ = 5.3625, β = − 1.67; R2 p = 0.0111, F_1,7.9_ = 10.869, β = − 2.22; R3 p = 0.0054, F_1,7.7_ = 14.762, β = − 2.5). Ribs R1, R2 and R3 were shorter in pelvic brooding compared to transfer brooding species (Fig. 3, Additional file 1: Table S1). Additionally, we found a significant interacting effect of reproductive strategy and sex on the length of ribs R2 and R3 (LMM: R2 p = 0.0107, F_1,21.3_ = 7.8324, β = 0.97; R3 p = 0.0009, F_1,21.7_ = 14.835, β = 0.89), indicating shorter ribs R2 and R3 in females of pelvic brooding species. Linear mixed models for rib height revealed similar patterns to rib length for most ribs (Additional file 1: Table S2). LMMs for ribs R-1 to R5 showed a significant correlation of rib height with reproductive strategy with shorter ribs in pelvic brooding species. Additionally, reproductive strategy and sex had a significant interacting effect on rib height in R2 to R4, pointing towards shorter ribs in pelvic brooding females (Additional file 1: Table S2). We further calculated whether each consecutive rib of ribs R-2 to R5 increased or decreased in length compared to the previous rib. In pelvic brooding females the first rib behind the pelvic fin insertion (R1) was consistently shorter than R0 (*A. oophorus:* -20.53%; *O. eversi:* -6.58%; *O. sarasinorum* -18.31%; Additional file 2: Figs. S1, S2). In contrast, in females of transfer brooding species R1 was slightly longer than R0: *O. celebensis*: + 0.95%; *O. matanensis*: + 7.79%; *O. nigrimas*: + 1.68%; *O. wolasi*: + 0.37% (Additional file 2: Fig. S2). From R1 to R2 ribs were more shortened in pelvic brooding species (*A. oophorus*: − 16.63%; *O. eversi*: − 16.70%; *O. sarasinorum*: − 23.06%) compared to transfer brooding species (*O. celebensis*: + 0.35%; *O. matanensis*: − 6.96%; *O. nigrimas*: − 2.25%; *O. wolasi*: − 1.70%). From R3 to R5, ribs of all investigated female specimens decreased in size, but ribs of pelvic brooding specimens were consistently shorter than those of transfer brooding species (see Fig. 3; Additional file 2: Fig. S2). Differences in the length of consecutive ribs located posterior of the pelvic girdle were also found in male pelvic brooding and transfer brooding species, but these were less pronounced than in females (Fig. 3). From R0 to R1, the change in rib length for male specimens of *A. oophorus, O. eversi* and *O. sarasinorum* was + 0.68%, -5.48% and -5.45%, respectively and for *O. celebensis*: − 0.19%; *O. matanensis*: + 4.35%; *O. nigrimas*: + 2.29% and *O. wolasi*: − 1.6% (Additional file 2: Fig. S3). Irrespective of the brooding strategy, rib R2 of males differed only slightly in length from R1: *A. oophorus*: − 4.02%; *O. eversi*: − 3.17%; *O. sarasinorum*: + 5.58%; *O. celebensis*: + 0.19%; *O. matanensis*: + 8.94%; *O. nigrimas*: + 1.78%; *O. wolasi*: − 1.6% (Additional file 2: Fig. S3). From R3 to R5, ribs of all males were consistently shorter, but no difference in relative rib length was present between pelvic brooding and transfer brooding males (Additional file 2: Fig. S3). The distance between the tips of each rib-pair (rib gap) became shorter from anterior to posterior across all studied specimens indicating that the rib cage gets narrower towards the anal fin (Additional file 2: Fig. S4).

In contrast to rib length or rib height, LMMs did not reveal any significant correlation between rib gaps caudal of the pelvic girdle and reproductive strategy (Additional file 1: Table S3).

### Pelvic fins are elongated and thickened in pelvic brooding and elongated in some transfer brooding females

**Fig. 4:**
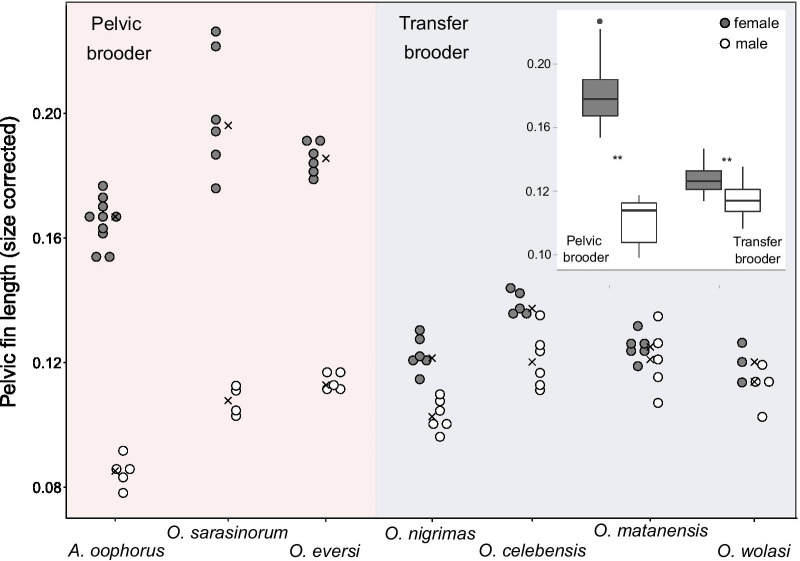
Differences in pelvic-fin lengths between pelvic and transfer brooding species. Fin length was corrected for individual standard length and measured in N = 77 specimens. Crosses represent within-group median. The boxplot in the top right corner shows the differences in fin length between the two reproductive strategies and sexes. Both comparisons are significant (indicated by the two asterisks)

Standard-length-corrected measurements of pelvic fin lengths for females and males of seven ricefish species revealed sex and brooding strategy dependent differences (Fig. 4, one-way ANOVA: F_13,63_ = 68.43 p < 0.0001, data were log_2_ transformed to meet normality). When pooling pelvic brooding and transfer brooding species respectively, comparisons in pelvic fin length were significant (one-way ANOVA: _F3,73_ = 116.7 p < 0.0001). In both reproductive strategies females had significantly longer pelvic fins than males (Fig. 4; boxplots, Tukey-HSD: p = 0.017 transfer brooding species, p < 0.0001 pelvic brooding species).

Females of most ricefish species had-independent of their reproductive strategy- significantly longer pelvic fins than their respective males (Wilcoxon-rank-sum test: *A. oophorus:* W = 50, p = 0.0007, 94.94% longer; *O. eversi*: W = 30, p = 0.004*,* 63.98% longer, *O. sarasinorum*: W = 24, p = 0.009*,* 85.82% longer, *O. nigrimas*: W = 36, p = 0.002, 20.04% longer, *O. celebensis*: W = 30, p = 0.004, 13.9% longer). We found no significant differences in fin length between the sexes of the transfer brooding species *O. matanensis* and *O. wolasi* (Wilcoxon-test: W = 18, p = 0.66 and W = 9, p = 0.4, respectively; Fig. 4). Overall, females of all pelvic brooding species had significantly longer pelvic fins compared to females and males of all transfer brooding species (Tukey-HSD: all p ≤ 0.001; Table 1, lower diagonal, Additional file 1: Table S4, Fig. 4). Among the pelvic brooding species, females of the two *Oryzias* species had significantly longer pelvic fins than female *Adrianichthys oophorus* (Fig. 4, Table 1) and among the transfer brooding species *O. celebensis* had significantly longer pelvic fins than *O. nigrimas* and *O. wolasi* females (Tukey-HSD: p = 0.025 and p = 0.035 respectively).

**Table 1:**
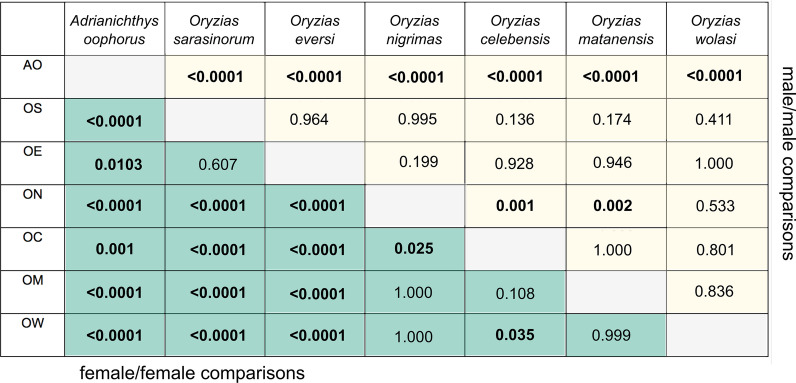
Post hoc comparisons of the Tukey-HSD-test for inter-specific differences in pelvic fin length

Female–female comparisons are shown in the lower diagonal and male–male comparisons in the upper diagonal. All p values adjusted for multiple testing. Significant comparisons are displayed in bold. AO = *Adrianichthys oophorus*, OS = *Oryzias sarasinorum*, OE = *O. eversi*, ON = *O. nigrimas*, OC = *O. celebensis*, OM = *O. matanensis*, OW = *O. wolasi*

The total cross sectional area (CSA) for the lateral (lfr) and the medial (mfr) measurement points, our estimator for the thickness of the respective pelvic fin ray, was largest in pelvic brooding females (Additional file 2: Fig. S5). The CSAs of males of pelvic brooding species measured only about one-third the size of their female conspecifics and resemble the lower CSA also found in both sexes of transfer brooding species (Additional file 1: Table S5, CSA column). CSA, i.e. pelvic fin ray thickness, strongly depended on pelvic fin length. LMMs for the lateral measurement point revealed a significant correlation of fin ray thickness with fin length (LMM: p < 0.0001, F _1,11_ = 84.1493, β = 0.72) and sex (LMM: p = 0.0451, F _1,7.6_ = 5.7438, β = 0.25), indicating thicker lateral pelvic fin rays with increasing pelvic fin length and within female specimens (Additional file 1: Table S6). In the medial fin ray, a significant interaction of reproductive strategy and sex on pelvic fin thickness (LMM: p < 0.0001, F _1,7.8_ = 164.7435, β = − 1.35) was evident, pointing towards the thinner medial fin rays of male pelvic brooders. In the medial fin ray we didn´t find a significant correlation of fin length and fin thickness, however standard length was significantly correlated (LMM: p < 0.0183, F _1,13.8_ = 7.1616, β = 0.22). When corrected for fin length, the relative fin ray diameter of the lateral and medial fin ray was lower in female specimens of pelvic brooding species when compared to their conspecific males. This is also true in transfer brooding species, except for *O. celebensis* (Additional file 1: Table S5).

### In contrast to transfer brooders, females of pelvic brooding species have smaller body cavities than their males

As LMMs revealed that SL has the largest effect on approximated body cavity volumes (Additional file 1: Table S7), we present differences between and within species based on ratios corrected for standard length (Table 2). Statistics, however, are only presented based on LMMs including ‘standard length’ as covariate.

**Table 2:**
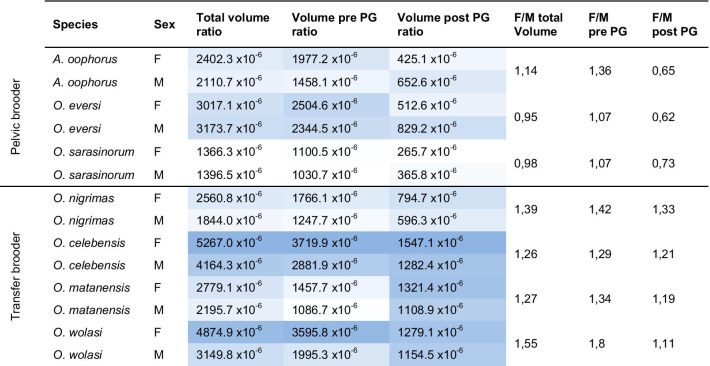
Body volumes corrected for standard length

Volumes for female ricefishes are averaged for the two or three replicates of each species. Intensity of color shades indicates smaller (more white) to larger (more blue) values for each column. *PG* pelvic girdle, *F* female, *M* male

Generally, size-corrected body cavity volumes varied considerably across species (Table 2, total volume) thus not systematically differentiating between pelvic brooding and transfer brooding species. However, females of all transfer brooding species had larger size-corrected body cavities than their conspecific males. In contrast, females of the pelvic brooding species *O. eversi* and *O. sarasinorum* had smaller body cavities than their conspecific males (Table 2, F/M total Volume). A significant effect of sex on total body cavity volume was also supported by the LMM for body volume (p_sex_ = 0.0001, F_1, 6.15_ = 71.52, β = − 0.44, Additional file 1: Table S7).

To test whether a potential reduction of available space due to the ventral concavity is compensated for, we divided the body in two segments: pre- and post-pelvic girdle. LMMs revealed a significant interaction of sex and reproductive strategy on the post pelvic girdle volume (p_sex:type_ = 0.0023, F_1,6.57_ = 23.2881, β = 0.63), but not on the pre pelvic girdle volume (Additional file 1: Table S7). The size-corrected pre-pelvic girdle volume (Table 2, F/M pre-PG) was larger in females of all species than in males, but in the post pelvic girdle segment the volume of pelvic brooding females was smaller than in conspecific males and transfer brooding females (Table 2, F/M post-PG). Here, female to male ratios in cavity volumes in pelvic brooding species amounted to only about half of those of transfer brooding species (Table 2, F/M post PG). In the pelvic brooding *A. oophorus*, which form the sister group to the remaining studied species, body volume anterior to the pelvic girdle (F/M ratio) was similar to that of transfer brooding species, but lowest (F/M ratio) posterior to the pelvic girdle.

### Phylogenetic signal does not explain skeletal differences between reproductive strategies

To evaluate phylogenetic effects, we used phylogenetic generalized least square analyses (PGLS). Maximum likelihood ratio tests (LRTs) were used to estimate λ. LRTs indicated phylogenetic independency (λ = 0) of rib length and rib height regardless of sex, with the exception of one rib in males (R-2, rib length) (Additional file 3: Sheet S1 and S2). For ribs R1–R4 (rib length) and ribs R1-R3 (rib height) in females, λ was significantly different from 1 and PGLS confirmed significant differences in rib length (R2 and R3) and rib height (R1–R5) between females of pelvic brooding and transfer brooding ricefishes (Additional file 3: Sheet S1 and S2). When setting λ = 1, PGLS for rib height (R2-R4) remained significantly different between female pelvic and transfer brooders (Additional file 3: Sheet S2). The degree of sexual dimorphism (ratio male/female) in rib height of R1-R4 differed significantly between both reproductive strategies. Differences between females of both reproductive strategies were confirmed for the medial pelvic fin ray area (mfr), which persisted under the assumption λ = 1. Lambda was significantly different from 1 for body cavity volumes posterior of the pelvic fin insertion, which differed significantly between females of pelvic brooders and transfer brooders. Similarly, the male/female ratio of both, the total body cavity volume and the post-pelvic fin insertion volume differed between both reproductive strategies (Additional file 3: Sheet S6). While lambda was not significantly different from 1, significant differences in sexual dimorphism of the pre-pelvic fin body cavity volumes between both reproductive strategies persisted under the assumption λ = 1 (Additional file 3: Sheet S6).

## Discussion

### Brooding strategy and sex are linked to rib and pelvic fin morphology in Sulawesi ricefishes

Females of pelvic brooding species have shorter ribs located at and caudal to the pelvic girdle, as well as longer and thicker pelvic fins compared to conspecific males and both sexes of transfer brooding ricefishes. Particularly, thoracic ribs R1, R2 and R3 are shorter (Figs. 2, 3) and located where female pelvic brooders exhibit a concave dent termed ‘ventral concavity’ [28]. The ventral concavity is a female specific adaptation to pelvic brooding, as females situate the egg-cluster inside this concavity during the prolonged brooding period. This was first reported for lake-dwelling *A. oophorus* [28] and later also described in the two other pelvic brooding species, the lake-dwelling *O. sarasinorum*, and *O. eversi*, which inhabits a small karst pond [24, 29]. Underlying skeletal adaptations of the ventral concavity were not systematically analyzed so far, and we show that shorter thoracic ribs closely behind the pelvic girdle create the concave dent in female pelvic brooding ricefishes. Such sex-specific modifications linked to a species reproductive ecology are generally rare. In banded-darter dragonflies it was shown that sexually dimorphic wing shape was likely shaped by different flight requirements in tandem flights or migration [37]. Bill morphology in birds can be dimorphic when males and females pursue different foraging strategies, as shown for hermit hummingbirds [38]. Dimorphic modifications of the ribs are uncommon among vertebrates. Among the few examples for fishes are the deep-sea fish *Barathrodemus manatinus* (Ophidiidae) and the miniature freshwater swamp specialists of the genus *Paedocypris*. In the former, males lack the first anterior rib, creating space for enlarged drumming muscles that likely produce a sound to attract conspecific females (e.g. [39]). *Paedocypris* exhibit a pronounced sexual dimorphism in their skeleton that also includes ribs, which is supposed to be related to reproduction (e.g. [40]).

Adaptations of the pelvic fins including their reductions or complete loss are more common in teleost fishes [41]. Thus it appears plausible, that pelvic fins are less important for swimming than other fins [42, 43], potentially relaxing natural selection acting upon them [44]. Extreme examples of such modifications are the adhesive sucking disks in gobies [45], wings for short-term flight in flying fishes [46], specialized brood pouches in female ghost pipefishes [47] or elaborate copulatory organs, like the priapium in male priapium fish [48, 49]. In ricefishes, lower evolutionary constrains might have favored the evolution of thickened and elongated pelvic fins in females of pelvic brooding species, contrasting the common form of shorter pelvic fins observed in all other ricefishes. Variation in pelvic fin length and thickness were already described for *O. sarasinorum* in 1905 by [31] but without recognizing the correlation with the respective sex or brooding strategy of the specimens. Since then, sexual dimorphism in pelvic fin length was well described for all pelvic brooding species [24, 27, 50], but was only recently recognized in two transfer brooding species [33, 34]. In the present study we found longer pelvic fins in females of two additional transfer brooding species (*O. celebensis*, *O. nigrimas*) indicating that sexual dimorphism in pelvic fins, with females having longer fins, is a reoccurring pattern in ricefishes. The sex-specific size difference was, however, much smaller among transfer brooding compared to pelvic brooding species, with pelvic fins of pelvic brooding females reaching up to twice the size of fins of conspecific males whereas in transfer brooding species female pelvic fins were only around one-third longer (Fig. 4). Comparatively long and thick pelvic fins seem to be crucial in the context of pelvic brooding, but may also be advantageous for some transfer brooding females. The heterogeneity of this trait in transfer brooding species might relate to potential variation in the duration of egg carrying which could be associated with environmental settings [28, 51–53]. Data, however, remain scarce and further research is needed to shed light on the impact of general ecology on reproductive biology of ricefishes.

### Adaptations to pelvic brooding evolved in parallel in two lineages of ricefishes and likely come at a cost

In Sulawesi’s pelvic broodings ricefishes, repeated adaptation to similar brooding strategies resulted in the evolution of similar skeletal traits in two distantly related lineages. Correcting for phylogenetic signal or assuming strong phylogenetic effect did not change our results, despite low sample size and thus lower statistical power of the models (Additional file 3; [54]), i.e., similar parallel adaptations to pelvic brooding most likely shape the examined skeletal traits. Pelvic brooding likely evolved rather recently in the stem lineage of *O. sarasinorum/eversi* (Fig. 1), indicating that several characteristic adaptations to this novel reproductive strategy evolved within a relatively short period of time ([22]; ~ 1–1.5 myr). Up to the discovery of *O. eversi* in a small karst pond [24], pelvic brooding was assumed to be an adaptation to the open water habitats of Lakes Poso and Lindu [23, 28]. Given the distinct environmental settings pelvic brooding species occur in, selection pressures mediating pelvic brooding are likely not primarily related to macro-habitat conditions like pelagic lifestyle, but to small-scale factors, like predation or interspecific competition. Provisioning care often comes at the cost of increased predation risk [55, 56], reduced feeding opportunities [57, 58], retarded growth and reduced fecundity [12] of the care-giver. In contrast to daily spawning transfer brooding ricefish species, females of pelvic brooding species carry a bundle of ~ 20–30 eggs [27] between 11 up to 18 days at 25–27 °C ([27], own aquaria observation). Skeletal adaptations that reduce costs of egg carrying, e.g. by improving hydrodynamics during brooding, are thus only expected to be advantageous for female pelvic brooders in the context of maternal care. Indeed, elongated pelvic fins that cover the entire egg cluster in combination with shortened ribs are only found in female pelvic brooders, indicating sexually antagonistic selection in pelvic brooding species [59]. Additionally, transfer brooding species mostly exhibit distinct or less pronounced trait characteristics, which underlines that these traits are likely maladaptive for ricefishes not providing care in the form of pelvic brooding, e.g. the ventral concavity is not present in any other ricefish species with known reproductive strategy [23].

### Ecological rather than sexual selection shapes female-specific traits

While most sexually dimorphic traits evolve under sexual selection, it appears rather unlikely that sexual selection [60] plays a major role for the maintenance of the observed sexual dimorphism in pelvic brooding ricefishes. Studies on sexual selection in ricefishes are rare and focus on Medaka (*O. latipes*). In Medaka, sexual selection primarily acts via male-male competition and female preference for elongated dorsal and anal fins in males [61], even though one study also shows that male *O. latipes* prefer larger females [62]. For pelvic brooding species no data on mate choice is available. Even though it cannot be completely ruled out that males also choose mates, the much higher female investment makes it seem unlikely that males are the choosy sex. Hence, we assume that the effect of ecological selection pressures (e.g. on swimming performance in females) on the sexually dimorphic traits in pelvic brooding ricefishes is higher than sexual selection pressures. Similar examples, where sexual dimorphisms arise that are not primarily shaped by sexual selection or restricted to reproductive or copulatory organs are rare [11, 38]. However, evidence exists that the wing shape in female bats [63] and flying lizards [64] can be adapted to account for a sex-specific weight distribution during pregnancy. Also, sex-specific limb and trunk morphologies in wall lizards are likely adapted to specific locomotion needs [65] and larger skulls in female voles can improve their digging abilities and compensate for higher nutrition demands [66]. A comparable scenario in teleosts was recently discovered in cichlids, where sexual dimorphism in gill-raker length repeatedly evolved in uniparental mouthbrooders. This dimorphism evolved in response to contrasting demands of mouthbrooding on the one hand and trophic requirements on the other [15].

### Sex reversal in body cavity volume limits reproductive capacities

Body shape and size can be influenced by a variety of ecological factors, including habitat structure and sex [67–69]. The habitat of the four investigated transfer brooding species differs as they live in streams (*O. celebensis, O. wolasi*; [52, 53]) or the littoral zone of lakes (*O. nigrimas, O. matanensis*; [28, 51]). They all exhibit distinct body shapes and body cavity volumes, e.g. between the deep-bodied riverine *O. celebensis* and the slender lacustrine *O. nigrimas* (Table 2, Fig. 2). Despite this, body cavity volumes are consistently larger in transfer brooding females, than in their conspecific males (Table 2, Volume pre/post PG). In contrast, females of the pelvic brooding species *O. eversi* and *O. sarasinorum* have smaller body cavities than their conspecific males (Table 2, F/M total Volume) and all female pelvic brooders have a reduced body volume in the abdominal region due to the presence of the ventral concavity. Uneven parental investments likely create distinct trait optima concerning body shape and cavity volumes, e.g. as females need space for growing oocytes [26, 70]. We find this reflected in transfer brooding species, where female body cavity volumes are larger, but not in pelvic brooding species, where females even have smaller body volumes than males. There are no hints for a compensation of this loss in *O. eversi* and *O. sarasinorum*, e.g. by increasing volume anterior to the ventral concavity, but interestingly we see it in *A. oophorus*. Here, females possess a comparatively larger body cavity volume anterior to the ventral concavity and seem to at least partly compensate for the loss of volume at the ventral concavity (Table 2).

### Skeletal adaptations to pelvic brooding might improve hydrodynamics during brooding and may help to protect the eggs

Females of pelvic brooding species all exhibit a ventral concavity accompanied by clearly elongated and thickened pelvic fins and occur in very distinct habitats. Thus, this trait combination is likely beneficial in the context of the common reproductive strategy—pelvic brooding. It could for example allow for the creation of a more streamlined shape, as female pelvic brooders use the elongated pelvic fins to move the egg-cluster from the water stream into the ventral concavity while swimming. Hence, both ventral concavity and elongated pelvic fins in pelvic brooding ricefishes appear to be adaptations reducing drag during brooding. Furthermore, enlarged pelvic fins might help to mediate the likely negative hydrodynamic effects of the ventral concavity, when pelvic brooding females are not brooding. To cover the entire egg-cluster over the prolonged brooding period with elongated pelvic fins is likely beneficial, as it also provides protection from egg predators (conspecific and interspecific) and disturbing environmental factors (e.g. sources of mechanical damage). Moreover, egg-carrying females of pelvic brooding species occasionally move the eggs with their pelvic fins while resting in the water column (pers. aquarium observation), which might be aided by enlarged fins. Such a behavior resembles the movement of eggs as described for mouth-brooding cichlids, where the egg-carrying parent churns the eggs and young larva in their mouth [71]. Constant movement likely serves the aeration and cleaning of the eggs in cichlids appears to be crucial for normal development and egg survival [72]. It seems that, at least in the aquarium, eggs located on the margins of an egg cluster are more prone to become covered by algae and fungi. While moving the eggs might mechanically remove some of this biofilm and facilitate aeration, this hypothesis has to be investigated and remains speculative at this point. Ultimately, observations in the natural habitat will be required to disentangle potential drivers of female specific adaptations to pelvic brooding. Unfortunately, natural habitats of ricefishes in Sulawesi are massively disturbed by anthropogenic factors and invasive species, likely setting a time limit for observations in the wild. The population of pelvic brooding *Oryzias eversi,* for example, locally endemic to a small karst-pool in Tana Toraja, is presumably on the brink of extinction (personal observation).

Future studies should address the consequences of the amplified female investment in pelvic brooding ricefishes, which is expected to result in an increased female choosiness, which then might entail higher conspecific male-male competition [8]. Also, investigating costs and benefits of pelvic brooding in the context of the natural environment (e.g., including predation risk and habitat ecology) will provide more insight on how and why this complex maternal care mechanism evolved.

## Conclusions

We show that the two lineages of pelvic brooding ricefishes evolved extremely similar skeletal adaptations, which include shorter ribs and longer and thicker pelvic fin rays. The ventral concavity, which is present in female pelvic brooding species, causes a reduced body volume in the posterior region of the thorax, which is only partly compensated for. How exactly female-specific adaptations to pelvic brooding relate to their natural environment and ultimately transfer into a benefit remains to be investigated.

## Materials and methods

### Study specimens

The present study is based on seven species of ricefishes (Adrianichthyidae): *Oryzias eversi*, *O. nigrimas*, *O. sarasinorum*, *O. wolasi*, *O. celebensis, O. matanensis* and *Adrianichthys oophorus*. The selected species cover all six clades of endemic Sulawesi ricefishes according to [22] and include all three pelvic brooding species described to date [24, 28, 31, 73]. All samples belong to the ichthyology collection of the Zoological Research Museum Alexander Koenig (ZFMK) in Bonn, Germany or the Museum of Zoology (MZB) in Bogor, Indonesia. As sample availability differed among species and sexes, we provide details of all specimens investigated in the supplement (Additional file 1: Table S8). Raw data of measurements are provided in Additional file 4. 

### µ-CT imaging

For µ-CT imaging, we used 26 specimens comprising four adult individuals (3 × female; 1 × male) of each *Oryzias eversi*, *O. nigrimas*, *O. sarasinorum*, *O. wolasi* and *Adrianichthys oophorus*, and three individuals (2 × female; 1 × male) of *O. celebensis* and *O. matanensis*. Sample material for male specimens in suitable condition for µ-CT scanning was limited, so we could include only one male per species. Samples were transferred into polypropylene tubes and fully covered with 70% ethanol to prevent shrinkage from dehydration. To avoid movement during the imaging process, specimens were fixed with small polystyrene blocks. Full-body scans were obtained with a Bruker Skyscan 1173 computer tomographer, operating at 50–66 kV and 140–160 µA. The x-ray detector was set to an image resolution of 1120 × 1120 pixels (2 × 2 binning) and X-ray tube values were tuned for each specimen individually in order to adjust for optimal x-ray transmission intensities. For the analysis of pelvic fin rays, high-resolution images of 18 specimens were obtained with a Bruker Skyscan 1272 computer tomographer, operating at 50–56 kV and 160–166 µA. Image resolution was set to 2016 × 1344 pixels (2 × 2 binning) with an image pixel size of 12 µm. Additional file 1: Table S9 provides details for all scans. Subsequent image treatment and the computation of cross sections were carried out using Bruker’s DataViewer, NRecon and CTan software packages.

Full-body cross sections of all 26 individuals were transferred into the Drishti Import tool provided with the Drishti software package [74] to generate volume renders. All µ-CT scans are part of the collection of the Museum Koenig (Additional file 5).

### Ribs

**Fig. 5:**
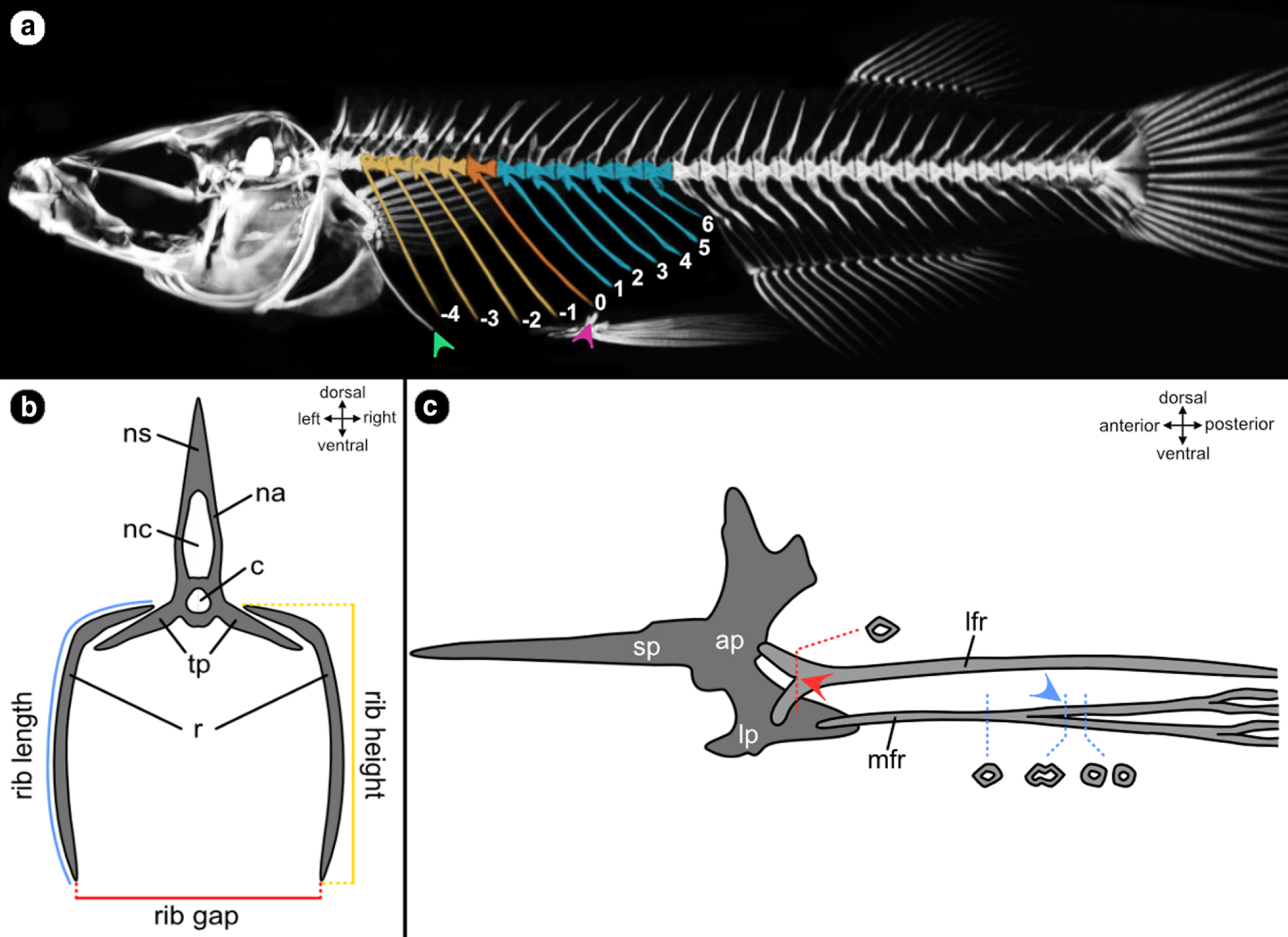
Overview of rib and pelvic fin measurements. **a** *O. eversi* skeleton with ribs numbered relative to rib R0 (orange), which lies above the insertion site of pelvic-fin rays into the pelvic girdle (purple arrow). Ribs anterior (yellow) of rib R0 received a negative, ribs posterior (blue) a positive value. The first rib (green arrow) was often incomplete and could not be measured reliably. It was thus not included. **b** Schematic cross-section of a vertebra including ribs illustrating rib length (blue), rib height (yellow) and rib gap (red). c = notochord, na = neural arch, nc = neural canal, ns = neural spine, r = rib, tp = transversal process. **c** Schematic view on the ventrolateral side of the pelvic girdle. The fin rays (light gray) insert into the articular plate (ap), to which a stick-like process (sp) and the lateral process (lp) are connected. Red and blue arrows indicate the sites were cross-section areas have been measured on the lateral fin ray (lfr) and medial fin ray (mfr)

Length measurements of individual ribs were performed by using the point-function in Drishti (version 2.7), placing landmarks onto the outermost voxels of the bony elements and later connecting them to form a line. To compare rib measurements across selected ricefish species, thoracic ribs were numbered depending on their location relative to the base of the lateral process [23] of each specimen’s pelvic girdle. As we were focusing on potential differences of rib lengths in the region of the pelvic fins where the egg-bundle is situated during brooding, the pelvic girdle was chosen as a reference point from where the ribs were numbered. Ribs aligned with this position were defined as rib 0 (R0; colored orange in Fig. 5a). Ribs anterior to it were numbered incrementally with negative numbers (colored yellow in Fig. 5a), ribs posterior were labeled with positive numbers (colored blue in Fig. 5a). Potentially incomplete ribs (e.g. caused by fracture or ontogenic reasons) [23] attached to the anterior most vertebrae were not counted.

Rib length (Fig. 5b) was defined as the length of the costal arch between its articulation at its vertebra and the ventral tip of a rib. We measured rib length by orienting the specimen’s volume-render with its head pointing left and orthogonally to the viewing plane. First, landmarks were placed centrally onto the bones from a dorsal viewing angle on the rib portion close to its respective vertebra; then the render was rotated 90° along the longitudinal body axis to mark the remaining thoracic part of each rib. Rib gap (Fig. 5b) was defined as the distance between the left and the right rib of the same vertebra. This measure was taken by placing points onto the tip of each rib and measuring the distance as a straight line between them. Finally, rib height (Fig. 5b) was defined as the maximum extension of the rib from the vertebral column independent from its curvature. It was measured by calculating the direct distance between the ventral tip and the dorsal end of a rib in maximum intensity projections from a lateral perspective using the measurement-tool embedded in Fiji [75].

### Pelvic-fins

As pelvic fins are generally enlarged in females of pelvic brooding species, we intended to investigate sexual dimorphism in pelvic fin length beyond pelvic brooding species as well as include a measure for pelvic fin thickness. We measured the length of the left pelvic fin using a digital caliper in 77 individuals of four transfer brooding species and three pelvic brooding species (*Oryzias wolasi*: N_fem_ = 3, N_male_ = 4; *O. matanensis:* N_fem_ = 6, N_male_ = 5; *O. celebensis:* N_fem_ = 5, N_male_ = 6; *O. nigrimas:* N_fem_ = 6, N_male_ = 6; *O. eversi:* N_fem_ = 6, N_male_ = 5; *O. sarasinorum:* N_fem_ = 6, N_male_ = 4; *Adrianichthys oophorus:* N_fem_ = 10, N_male_ = 5). Data were analyzed using pairwise tests and analysis of variance (ANOVA). For non-normally distributed data we used non-parametric pairwise tests and log_2_ transformation prior to the ANOVA. As a proxy for pelvic fin thickness, we calculated the cross sectional area of two positions in the lateral and a more medial pelvic fin ray based on high-resolution µ-CT images. The chosen locations are based on homologous points identified across all individuals, representing a fusion (lateral ray) and a bifurcation (medial ray) in the pelvic fin rays (Fig. 5c). High-resolution cross sections of 18 ricefish specimens (one individual for each species and sex with N = 3 females for *O. eversi* and *O. nigrimas*) were analyzed using Amira’s (Thermo Fisher Scientific, Version 6.5.0) segmentation tool. The measurement point (lfr) is located in the most lateral fin ray of each fin at its base where two hemitrichs fuse into one ray (Fig. 5c, red arrow head). The second measurement point (mfr) is located in the third medial fin ray, where the fin ray divides into two distal branches (Fig. 5c, blue arrow head).

For each specimen, the section of the µ-CT scan that depicts the lateral measurement point in the left fin was located. A grey-value-based selection was performed to mask the bony portion and the intra-ray space of the fin ray. Using the quick-selection tool in Amira we then used the same grey value threshold to also mask the corresponding measurement point in the right fin. This process was repeated for the medial measurement point and for all 18 specimens. Subsequently, each selection was assigned to a new layer in the Amira segmentation editor and the surface area of each layer was measured. For further analyses, mean values between left and right fin and for species represented by more than one individual (*O. eversi* and *O. nigrimas*) were taken. To correct for varying pelvic fin length among ricefish species and to test for disproportionately thicker pelvic fins rays, we also calculated the diameter from the areas of the lfr and mfr measurement points, which are more or less circular, and divided the diameter by each specimen’s pelvic fin length.

### Body cavity volumes

We estimated the abdominal cavity volume of 26 ricefish specimens, using the length of the body cavity, ‘rib height’ and ‘rib gap’. The length of the body cavity was measured in the previously obtained maximum intensity projections from the anterior part of the first vertebra to the tip of the first haemal spine using the software package Fiji. To evaluate the effect of the ventral concavity on the body cavity volume in the anterior and posterior regions of the body, body cavity volumes were calculated separately for the region anterior of the pelvic girdle (pre-pelvic girdle) and posterior of the pelvic girdle (post-pelvic girdle), where the ventral concavity is situated. The pre-pelvic girdle segment ranged from the most anterior vertebra with an attaching rib to the insertion of the pelvic fin rays into the pelvic girdle. The post-pelvic girdle segment ranged from this insertion site to the first interhaemal bone of the anal fin.

We accounted for the effect of body size in the relative volumes, by dividing rib heights, rib gaps and the lengths of the body cavities by each specimen’s standard length, i.e., the distance between the tip of the snout and the caudal peduncle. For rib height and rib gap, we calculated means for the pre- and post-pelvic girdle segment for each species. Both means were then multiplied with the size-corrected body cavity length of each segment to generate a relative measure of the body cavity volume in relation to body size. This measure served as an approximation of the maximum available abdominal volume relative to the body size. In linear mixed models, absolute values for rib height, rib gap and length of body cavity sections were used to calculate approximated volumes and standard length was included as explanatory variable. Ricefish species differ in their ecology, which may affect body sizes and shapes and thus their relative body cavity volumes independent of their reproductive strategy. Hence, to evaluate whether pelvic brooding is associated with a loss in relative body cavity volume, we used the mean female to male body cavity volume ratios for each species. The use of this measure is based on the assumption that overall ecology and body shape are more similar between the different sexes of the same species than between the same sexes of different species. A reduced body cavity volume caused by the evolution of the ventral concavity in pelvic brooding females would thus be reflected in a smaller female to male body cavity ratio in pelvic brooding species compared to the same ratio in transfer brooding species. If driven by the ventral concavity, we expected this difference to be particularly pronounced in the post-pelvic girdle volume.

### Statistical analyses

Linear mixed effect models were conducted in R using ‘fit linear mixed-effects models’ (lmer). LMMs were created using the ‘lme4′ package. The models were used to test the effect of sex, reproductive strategy and standard length (explanatory variables) on rib length, rib height, rib gap, pelvic fin ray thickness and body volume (dependent variables). The factor ‘species’ was added as a random factor. As adaptations to pelvic brooding are likely biased towards the caring sex, we allowed an interaction between the explanatory variables reproductive strategy and sex. For rib length, rib height and rib gap, each rib or rib pair was tested separately. Because the number of ribs varies between different ricefish species, only measurements for ribs present in all individuals (R-2 to R5, Fig. 5a) were included. For R-2 (rib length) and R5 (rib gap) the initial models estimated variances of zero for the random effect ‘species’, indicating an overfitted model. Therefore, we adjusted the model for these ribs by reducing the complexity, i.e. removing the random effect, to obtain a better-fit model [76].

For models on pelvic fin ray thickness, we additionally included ‘fin length’ as explanatory variable to account for potential allometric effects in the pelvic fin. For the LMM analysis we calculated means from the left and right pelvic fin for both individual measurement points (lfr and mfr, Fig. 5c).

The function ‘step’ of the ‘lmerTest’ package [77] was used for all models to perform a backward elimination of non-significant effects of linear mixed-effect models. Ultimately, this ‘step’ process continues until the variables that best explained the model are found to provide significance levels for the predictor variables based on F-statistics. Subsequently, the residuals of the best fitting model were tested for normal distribution. As residuals of the models for fin ray area (lfr, mfr) showed significant deviation from normal distribution (Shapiro–Wilk test, W_lfr_ = 0.89, p = 0.002; W_mfr_ = 0.92, p = 0.016) a log_2_ transformation was conducted on raw data for lfr and mfr to obtain normal distributions and models were repeated as described above. To further interpret our results regarding sex or brooding strategy dependent differences, we calculated standardized (β) coefficients for the explanatory variables to estimate the effect size of each predictor using the effectsize package for R [78].

For the analysis of pelvic fin length, we used two tailed t-tests or non-parametric Wilcoxon rank sum tests, depending on normality of the data. Tests for normality and homogeny were carried out in R (v.3.6.3).

In order to assess the impact of phylogenetic effects in our dataset, we conducted a phylogenetic generalized least square (PGLS) analysis based on a published ricefish phylogeny [79] using the pgls function in the R (version 4.0.3) package ‘caper’ [80]. Using the drop.tip function of the R package ‘ape’ [81], we pruned the ricefish phylogeny to only contain the seven species included in our study. To match the tips of the phylogeny, we tested each sex separately and calculated species-specific mean values for all traits for males and females separately. A male-to-female ratio was calculated for each trait, to test differences in the degree of sexual dimorphism between reproductive strategies. All models included the trait of interest as dependent variable, ‘reproductive strategy’ as predictor and ‘standard length’ as covariate. The PGLS analysis was conducted in two steps: first, the scaling parameter λ was calculated using likelihood ratio tests (LRTs) to assess the degree of phylogenetic independence of our data [82]. λ varies between 0 and 1; values of λ = 0 indicate phylogenetic independence of traits while values of λ = 1 indicate that traits evolved according to Brownian motion [82]. For traits, where λ did not significantly differ from 1 λ was set to 1. Since low species numbers limit the power to estimate λ [54], we ran PGLS with λ set to 1 for all traits in which lambda did not differ significantly from 1. By doing this, we got an estimate, which traits are affected by phylogenetic effect and if a phylogenetic signal could not be rejected, we tested how our results change under the assumption of a strong phylogenetic effect.

## Supplementary Information

**Additional file 1.** Additional tables.

**Additional file 2.** Additional figures.

**Additional file 3.** The PGLS results for the phylogenetic models.

**Additional file 4.** Raw data used for analyses presented in the main manuscript.

**Additional file 5.** Collection numbers of used specimens and corresponding CT datasets.

## Data Availability

The µ-CT scans generated and analyzed in this study are available via the Morphobank project 3816 (http://morphobank.org/permalink/?P3816).
