# Supplementary material for: Complex sexually dimorphic traits shape the parallel evolution of a novel reproductive strategy in Sulawesi ricefishes (Adrianichthyidae)
Source: BMC Ecol Evol. 2021 Apr 20;21:57. doi: 10.1186/s12862-021-01791-z (PMC8056572; doi:10.1186/s12862-021-01791-z)
Supplement: Supplementary file 1 — Additional file 1. Additional tables. [file 12862_2021_1791_MOESM1_ESM.docx]

**Additional file 1 for: Complex sexually dimorphic traits shape the parallel evolution of a novel reproductive strategy in Sulawesi ricefishes (Adrianichthyidae).**

Tobias Spanke^1^, Leon Hilgers^1^, Benjamin Wipfler^1^, Jana M. Flury^1^, Arne W. Nolte², Ilham V. Utama³, Bernhard Misof^1^, Fabian Herder^1^ & Julia Schwarzer^1*^

^1^ Zoologisches Forschungsmuseum Alexander Koenig, Adenauerallee 160, 53113 Bonn, Germany

^2^ Carl von Ossietzky Universität Oldenburg, AG Ökologische Genomik, Carl von Ossietzky-Str. 9-11 26111 Oldenburg

^3^ Ichthyology Laboratory, Indonesian Institute of Sciences (LIPI), JL. Raya Jakarta-Bogor Km. 46, Cibinong 16911, Indonesia

- Corresponding author: Julia Schwarzer

**Additional file 1: Supplementary Tables**

**Table S1 Linear mixed effect model summary for rib length.**

| **R-2** |  |  |  | |  | |  | |  |  | |
| --- | --- | --- | --- | --- | --- | --- | --- | --- | --- | --- | --- |
| **Fixed effect** |  |  | *DF* |  | | *F* | | *β* | | *P* | |
| Standard length | |  | 1 |  | | 112.8224 | | 0.7154 | | **<0.0001** | |
| Sex |  |  | 1 |  | | 16.111 | | -0.4391 | | **0.0006** | |
| Reproductive strategy | | | 1 |  | | 3.5053 | | 0.5507 | | 0.0752 | |
| Sex*Reproductive strategy | | | 1 |  | | 2.536 | | -0.5713 | | 0.1262 | |
| **R-1** |  |  |  |  | |  | |  | |  | |
| **Fixed effect** |  |  | *numDF* | *denDF* | | *F* | | *β* | | *P* |  |
| Standard length | |  | 1 | 6.6209 | | 43.2823 | | 0.9081 | | **0.0004** | |
| Sex |  |  | 1 | 17.9858 | | 9.4855 | | -0.3038 | | **0.0065** | |
| Reproductive strategy | | | 1 | 5.758 | | 0.4044 | | -0.0033 | | 0.5493 | |
| Sex*Reproductive strategy | | | 1 | 19.3933 | | 2.9098 | | -0.5848 | | 0.104 | |
| **Random effect** |  |  |  |  | |  | |  | |  | |
| Species |  |  | 1 |  | |  | |  | | 0.0766 | |
| **R0** |  |  |  |  | |  | |  | |  | |
| **Fixed effect** |  |  | *numDF* | *denDF* | | *F* | | *β* | | *P* | |
| Standard length | |  | 1 | 8.0641 | | 31.0709 | | 1.103 | | **0.0005** | |
| Sex |  |  | 1 | 17.2137 | | 19.3509 | | -0.2041 | | **0.0004** | |
| Reproductive strategy | | | 1 | 6.2841 | | 4.2349 | | -0.458 | | 0.0832 | |
| Sex*Reproductive strategy | | | 1 | 19.8427 | | 7.4883 | | -0.7425 | | **0.0128** | |
| **Random effect** |  |  |  |  | |  | |  | |  | |
| Species |  |  | 1 |  | |  | |  | | **0.0013** | |
| **R1** |  |  |  |  | |  | |  | |  | |
| **Fixed effect** |  |  | *numDF* | *denDF* | | *F* | | *β* | | *P* | |
| Standard length | |  | 1 | 13.6221 | | 11.801 | | 1.0866 | | **0.0042** | |
| Sex |  |  | 1 | 19.2367 | | 0.5153 | | -0.2881 | | 0.4815 | |
| Reproductive strategy | | | 1 | 8.4103 | | 5.3625 | | -1.6706 | | **0.0477** | |
| Sex*Reproductive strategy | | | 1 | 21.5499 | | 1.045 | | 0.3551 | | 0.318 | |
| **Random effect** |  |  |  |  | |  | |  | |  | |
| Species |  |  | 1 |  | |  | |  | | **<0.0001** | |

**Table S1 (continued)**

| **R2** |  |  |  |  | |  | |  |  |  |
| --- | --- | --- | --- | --- | --- | --- | --- | --- | --- | --- |
| **Fixed effect** |  |  | *numDF* | *denDF* | *F* | | *β* | | *P* | |
| Standard length | |  | 1 | 10.091 | 9.5117 | | 0.7964 | | **0.0114** | |
| Sex |  |  | 1 | 18.9242 | 8.3505 | | -0.003 | | **0.0094** | |
| Reproductive strategy | | | 1 | 7.9018 | 10.869 | | -2.224 | | **0.0111** | |
| Sex*Reproductive strategy | | | 1 | 21.257 | 7.8324 | | 0.9697 | | **0.0107** | |
| **Random effect** |  |  |  |  |  | |  | |  | |
| Species |  |  | 1 |  |  | |  | | **0.0004** | |
| **R3** |  |  |  |  |  | |  | |  | |
| **Fixed effect** |  |  | *numDF* | *denDF* | *F* | | *β* | | *P* | |
| Standard length | |  | 1 | 15.459 | 18.32 | | 1.0148 | | **0.0006** | |
| Sex |  |  | 1 | 18.2563 | 19.126 | | 0.0234 | | **0.0004** | |
| Reproductive strategy | | | 1 | 7.6626 | 14.762 | | -2.4972 | | **0.0054** | |
| Sex*Reproductive strategy | | | 1 | 21.743 | 14.835 | | 0.8896 | | **0.0009** | |
| **Random effect** |  |  |  |  |  | |  | |  | |
| Species |  |  | 1 |  |  | |  | | **<0.0001** | |
| **R4** |  |  |  |  |  | |  | |  | |
| **Fixed effect** |  |  | *numDF* | *denDF* | *F* | | *β* | | *P* | |
| Standard length | |  | 1 | 16.999 | 3.0561 | | 0.585 | | 0.1012 | |
| Sex |  |  | 1 | 18.9895 | 14.3836 | | 0.2128 | | **0.0012** | |
| Reproductive strategy | | | 1 | 6.9856 | 2.3231 | | -1.7949 | | 0.1714 | |
| Sex*Reproductive strategy | | | 1 | 22.0634 | 3.0561 | | 0.4435 | | 0.09434 | |
| **Random effect** |  |  |  |  |  | |  | |  | |
| Species |  |  | 1 |  |  | |  | | **<0.0001** | |
| **R5** |  |  |  |  |  | |  | |  | |
| **Fixed effect** |  |  | *numDF* | *denDF* | *F* | | *β* | | *P* | |
| Standard length | |  | 1 | 13.3192 | 1.9879 | | 0.5376 | | 0.1815 | |
| Sex |  |  | 1 | 18.9722 | 15.1302 | | 0.2599 | | **0.001** | |
| Reproductive strategy | | | 1 | 6.9599 | 2.415 | | -1.7676 | | 0.1644 | |
| Sex*Reproductive strategy | | | 1 | 21.7206 | 3.6485 | | 0.5632 | | 0.0694 | |
| **Random effect** |  |  |  |  |  | |  | |  | |
| Species |  |  | 1 |  |  | |  | | **<0.0001** | |

Summary of the linear mixed effect models obtained with the lmerTest R package for rib lengths of R-2 to R5. Significance levels of p < 0.05 are highlighted in bold. Beta-coefficient reference for sex and reproductive strategy is ‘male’ and ‘pelvic brooding’ respectively.

**Table S2 Linear mixed effect model summary for rib height.**

| **R-2** |  |  |  |  |  |  |  |
| --- | --- | --- | --- | --- | --- | --- | --- |
| **Fixed effect** |  |  | *numDF* | *denDF* | *F* | *β* | *P* |
| Standard length | |  | 1 | 8.0225 | 15.6379 | 1.0946 | **0.0042** |
| Sex |  |  | 1 | 17.9731 | 3.5343 | -0.23 | 0.0764 |
| Reproductive strategy | | | 1 | 7.1386 | 3.8 | -0.8973 | 0.0915 |
| Sex*Reproductive strategy | | | 1 | 20.1382 | 0.3934 | -0.2310 | 0.5376 |
| **Random effect** | |  |  |  |  |  |  |
| Species |  |  | 1 |  |  |  | **0.0053** |
| **R-1** |  |  |  |  |  |  |  |
| **Fixed effect** |  |  | *numDF* | *denDF* | *F* | *β* | *P* |
| Standard length | |  | 1 | 10.8749 | 23.336 | 1.0638 | **0.0005** |
| Sex |  |  | 1 | 18.5552 | 5.7345 | -0.1735 | **0.0274** |
| Reproductive strategy | | | 1 | 7.4011 | 8.6960 | -1.256 | **0.0201** |
| Sex*Reproductive strategy | | | 1 | 21.2438 | 2.1212 | -0.4769 | 0.16 |
| **Random effect** | |  |  |  |  |  |  |
| Species |  |  | 1 |  |  |  | **0.0004** |
| **R0** |  |  |  |  |  |  |  |
| **Fixed effect** |  |  | *numDF* | *denDF* | *F* | *β* | *P* |
| Standard length | |  | 1 | 12.9498 | 18.5557 | 0.9825 | **0.0009** |
| Sex |  |  | 1 | 19.3343 | 0.0425 | 0.0293 | 0.8388 |
| Reproductive strategy | | | 1 | 8.3974 | 11.3364 | -1.6341 | **0.0092** |
| Sex*Reproductive strategy | | | 1 | 21.5975 | 0.1737 | -0.1318 | 0.6809 |
| **Random effect** | |  |  |  |  |  |  |
| Species |  |  | 1 |  |  |  | **0.0001** |
| **R1** |  |  |  |  |  |  |  |
| **Fixed effect** |  |  | *numDF* | *denDF* | *F* | *β* | *P* |
| Standard length | |  | 1 | 12.0509 | 6.4975 | 0.7472 | **0.0254** |
| Sex |  |  | 1 | 18.8999 | 4.6845 | 0.093 | **0.0435** |
| Reproductive strategy | | | 1 | 7.8621 | 10.9066 | -2.0401 | **0.0111** |
| Sex*Reproductive strategy | | | 1 | 21.7189 | 3.0745 | 0.5444 | 0.0936 |
| **Random effect** | |  |  |  |  |  |  |
| Species |  |  | 1 |  |  |  | **<0.0001** |

**Table S2 (continued)**

| **R2** |  |  |  |  |  |  |  |
| --- | --- | --- | --- | --- | --- | --- | --- |
| **Fixed effect** |  |  | *numDF* | *denDF* | *F* | *β* | *P* |
| Standard length | |  | 1 | 11.26 | 5.3449 | 0.4918 | **0.0407** |
| Sex |  |  | 1 | 19.0379 | 23.8895 | 0.1983 | **0.0001** |
| Reproductive strategy | | | 1 | 8.1323 | 15.9821 | -2.1762 | **0.0038** |
| Sex*Reproductive strategy | | | 1 | 21.639 | 9.9057 | 0.8217 | **0.0047** |
| **Random effect** | |  |  |  |  |  |  |
| Species |  |  | 1 |  |  |  | **<0.0001** |
| **R3** |  |  |  |  |  |  |  |
| **Fixed effect** |  |  | *numDF* | *denDF* | *F* | *β* | *P* |
| Standard length | |  | 1 | 13.724 | 7.2812 | 0.5305 | **0.0176** |
| Sex |  |  | 1 | 19.2226 | 36.5902 | 0.2688 | **<0.0001** |
| Reproductive strategy | | | 1 | 8.5203 | 20.1636 | -2.2343 | **0.0017** |
| Sex*Reproductive strategy | | | 1 | 22.158 | 9.9792 | 0.6703 | **0.0045** |
| **Random effect** | |  |  |  |  |  |  |
| Species |  |  | 1 |  |  |  | **<0.0001** |
| **R4** |  |  |  |  |  |  |  |
| **Fixed effect** |  |  | *numDF* | *denDF* | *F* | *β* | *P* |
| Standard length | |  | 1 | 14.995 | 4.4701 | 0.4269 | 0.0517 |
| Sex |  |  | 1 | 18.9755 | 40.3136 | 0.3222 | **<0.0001** |
| Reproductive strategy | | | 1 | 7.0955 | 7.958 | -2.1128 | **0.0254** |
| Sex*Reproductive strategy | | | 1 | 18.976 | 4.6512 | 0.579 | **0.0441** |
| **Random effect** | |  |  |  |  |  |  |
| Species |  |  | 1 |  |  |  | **<0.0001** |
| **R5** |  |  |  |  |  |  |  |
| **Fixed effect** |  |  | *numDF* | *denDF* | *F* | *β* | *P* |
| Standard length | |  | 1 | 17.5136 | 1.1415 | 0.365 | 0.3 |
| Sex |  |  | 1 | 18.9772 | 23.0317 | 0.3533 | **0.0001** |
| Reproductive strategy | | | 1 | 6.9604 | 6.7584 | -1.8909 | **0.0356** |
| Sex*Reproductive strategy | | | 1 | 22.0635 | 1.996 | 0.3345 | 0.1717 |
| **Random effect** | |  |  |  |  |  |  |
| Species |  |  | 1 |  |  |  | **<0.0001** |

Summary of the linear mixed effect models obtained with the lmerTest R package for rib heights of R-2 to R5. Significance levels of p < 0.05 are highlighted in bold. Beta-coefficient reference for sex and reproductive strategy is ‘male’ and ‘pelvic brooding’ respectively

**Table S3 Linear mixed effect model summary for rib gap.**

| **R-2** |  |  |  |  |  | |  |  | |  |
| --- | --- | --- | --- | --- | --- | --- | --- | --- | --- | --- |
| **Fixed effect** |  |  | *numDF* | *denDF* | *F* | | *β* | *P* | |  |
| Standard length | |  | 1 | 17.558 | 9.1039 | | 0.8131 | **0.0076** |  |  |
| Sex | | | 1 | 18.9658 | | 9.4551 | -0.6808 | **0.0062** | | |
| Reproductive strategy | | | 1 | 8.4612 | 0.0168 | | -0.4015 | 0.9 | |  |
| Sex*Reproductive strategy | | | 1 | 22.227 | 6.4054 | | 0.6424 | **0.019** | |  |
| **Random effect** | |  |  |  |  | |  |  | |  |
| Species |  |  | 1 |  |  | |  | **<0.0001** | |  |
| **R-1** |  |  |  |  |  | |  |  | |  |
| **Fixed effect** |  |  | *numDF* | *denDF* | *F* | | *β* | *P* | |  |
| Standard length | |  | 1 | 9.9335 | 4.1745 | | 0.5981 | 0.0685 | |  |
| Sex |  |  | 1 | 19.1174 | 2.1375 | | -0.5604 | 0.16 | |  |
| Reproductive strategy | | | 1 | 8.2175 | 0.1557 | | -0.1171 | 0.7032 | |  |
| Sex*Reproductive strategy | | | 1 | 21.8649 | 3.2754 | | 0.6474 | 0.0841 | |  |
| **Random effect** | |  |  |  |  | |  |  | |  |
| Species |  |  | 1 |  |  | |  | **<0.0001** | |  |
| **R0** |  |  |  |  |  | |  |  | |  |
| **Fixed effect** |  |  | *numDF* | *denDF* | *F* | | *β* | *P* | |  |
| Standard length | |  | 1 | 11.148 | 3.6813 | | 0.5699 | 0.081 | |  |
| Sex |  |  | 1 | 19.1143 | 0.6367 | | -0.6035 | 0.4347 | |  |
| Reproductive strategy | | | 1 | 7.0735 | 1.7745 | | -0.1569 | 0.2241 | |  |
| Sex*Reproductive strategy | | | 1 | 19.0941 | 3.5797 | | 0.91 | 0.0738 | |  |
| **Random effect** | |  |  |  |  | |  |  | |  |
| Species |  |  | 1 |  |  | |  | **<0.0001** | |  |
| **R1** |  |  |  |  |  | |  |  | |  |
| **Fixed effect** |  |  | *numDF* | *denDF* | *F* | | *β* | *P* | |  |
| Standard length | |  | 1 | 9.4648 | 5.6598 | | 0.5827 | **0.04** | |  |
| Sex |  |  | 1 | 18.7463 | 2.41 | | -0.5272 | 0.1373 | |  |
| Reproductive strategy | | | 1 | 7.8302 | 0.4372 | | 0.049 | 0.5274 | |  |
| Sex*Reproductive strategy | | | 1 | 21.8095 | 3.3183 | | 0.5967 | 0.0823 | |  |
| **Random effect** | |  |  |  |  | |  |  | |  |
| Species |  |  | 1 |  |  | |  | **<0.0001** | |  |

**Table S3 (continued)**

| **R2** |  |  |  |  |  |  |  | |  |
| --- | --- | --- | --- | --- | --- | --- | --- | --- | --- |
| **Fixed effect** |  |  | *numDF* | *denDF* | *F* | *β* | *P* | | |
| Standard length | |  | 1 | 7.3374 | 8.8352 | 0.4852 | **0.0196** | |  |
| Sex |  |  | 1 | 17.3708 | 0.8111 | -0.3891 | 0.3801 | |  |
| Reproductive strategy | | | 1 | 5.9155 | 1.6802 | 0.4023 | 0.2432 | |  |
| Sex*Reproductive strategy | | | 1 | 20.2992 | 1.7026 | 0.4873 | 0.2065 | |  |
| **Random effect** | |  |  |  |  |  |  | |  |
| Species |  |  | 1 |  |  |  | **0.002** | |  |
| **R3** |  |  |  |  |  |  |  | |  |
| **Fixed effect** |  |  | *numDF* | *denDF* | *F* | *β* | *P* |  |  |
| Standard length | |  | 1 | 7.075 | 12.7896 | 0.4833 | **0.0089** | |  |
| Sex |  |  | 1 | 18.1751 | 0.0551 | -0.3381 | 0.817 | |  |
| Reproductive strategy | | | 1 | 6.2576 | 1.9451 | 0.3167 | 0.2106 | |  |
| Sex*Reproductive strategy | | | 1 | 19.5835 | 3.1166 | 0.9012 | 0.0931 | |  |
| **Random effect** | |  |  |  |  |  |  | |  |
| Species |  |  | 1 |  |  |  | 0.1812 | |  |
| **R4** |  |  |  |  |  |  |  | |  |
| **Fixed effect** |  |  | *numDF* | *denDF* | *F* | *β* | *P* | |  |
| Standard length | |  | 1 | 6.9046 | 17.0903 | 0.5052 | **0.0045** | |  |
| Sex |  |  | 1 | 18.4053 | 0.0823 | -0.2987 | 0.7775 | |  |
| Reproductive strategy | | | 1 | 6.0326 | 2.3218 | 0.3 | 0.1781 | |  |
| Sex*Reproductive strategy | | | 1 | 19.098 | 2.2068 | 0.8703 | 0.1537 | |  |
| **Random effect** | |  |  |  |  |  |  | |  |
| Species |  |  | 1 |  |  |  | 0.7679 | |  |
| **R5** |  |  |  |  |  |  |  | |  |
| **Fixed effect** |  |  | *numDF* |  | *F* | *β* | *P* | |  |
| Standard length | |  | 1 |  | 35.8176 | 0.7412 | **<0.0001** | |  |
| Sex |  |  | 1 |  | 0.1923 | -0.4522 | 0.6655 | |  |
| Reproductive strategy | | | 1 |  | 0.3708 | -0.0315 | 0.5491 | |  |
| Sex*Reproductive strategy | | | 1 |  | 1.5905 | 0.736 | 0.2211 | |  |

Summary of the linear mixed effect models obtained with the lmerTest R package for rib gaps of R-2 to R5. Significance levels of p < 0.05 are highlighted in bold. Beta-coefficient reference for sex and reproductive strategy is ‘male’ and ‘pelvic brooding’ respectively.

**Table S4 Results of the Tukey-HSD post hoc test for species comparisons between males and females.**

| **Comparison** | | | | **Results Tukey-HSD** | | | |
| --- | --- | --- | --- | --- | --- | --- | --- |
| species | sex | species | sex | diff | lwr | upr | p adjusted |
| **AO** | **m** | **AO** | **f** | -0.2892 | -0.3359 | -0.2424 | **<0.001** |
| **OC** | **m** | **AO** | **f** | -0.1364 | -0.1805 | -0.0923 | **<0.001** |
| **OE** | **m** | **AO** | **f** | -0.1607 | -0.2074 | -0.1139 | **<0.001** |
| **OM** | **m** | **AO** | **f** | -0.1363 | -0.1830 | -0.0895 | **<0.001** |
| **ON** | **m** | **AO** | **f** | -0.2039 | -0.2480 | -0.1598 | **<0.001** |
| **OS** | **m** | **AO** | **f** | -0.1852 | -0.2357 | -0.1347 | **<0.001** |
| **OW** | **m** | **AO** | **f** | -0.1669 | -0.2174 | -0.1164 | **<0.001** |
| **AO** | **m** | **OC** | **f** | -0.2158 | -0.2698 | -0.1618 | **<0.001** |
| **OC** | **m** | **OC** | **f** | -0.0631 | -0.1148 | -0.0114 | **0.0050** |
| **OE** | **m** | **OC** | **f** | -0.0873 | -0.1413 | -0.0334 | **<0.001** |
| **OM** | **m** | **OC** | **f** | -0.0629 | -0.1169 | -0.0089 | **0.0092** |
| **ON** | **m** | **OC** | **f** | -0.1305 | -0.1822 | -0.0789 | **<0.001** |
| **OS** | **m** | **OC** | **f** | -0.1118 | -0.1691 | -0.0546 | **<0.001** |
| **OW** | **m** | **OC** | **f** | -0.0936 | -0.1508 | -0.0363 | **<0.001** |
| **AO** | **m** | **OE** | **f** | -0.3401 | -0.3918 | -0.2884 | **<0.001** |
| **OC** | **m** | **OE** | **f** | -0.1873 | -0.2366 | -0.1380 | **<0.001** |
| **OE** | **m** | **OE** | **f** | -0.2116 | -0.2633 | -0.1599 | **<0.001** |
| **OM** | **m** | **OE** | **f** | -0.1872 | -0.2389 | -0.1355 | **<0.001** |
| **ON** | **m** | **OE** | **f** | -0.2548 | -0.3041 | -0.2055 | **<0.001** |
| **OS** | **m** | **OE** | **f** | -0.2361 | -0.2912 | -0.1810 | **<0.001** |
| **Comparison** | | | | **Results Tukey-HSD** | | | |
| species | sex | species | sex | diff | lwr | upr | p adjusted |
| **OW** | **m** | **OE** | **f** | -0.2178 | -0.2729 | -0.1627 | **<0.001** |
| **AO** | **m** | **OM** | **f** | -0.1686 | -0.2203 | -0.1169 | **<0.001** |
| OC | m | OM | f | -0.0159 | -0.0651 | 0.0334 | 0.9971 |
| OE | m | OM | f | -0.0401 | -0.0918 | 0.0116 | 0.2987 |
| OM | m | OM | f | -0.0157 | -0.0674 | 0.0360 | 0.9983 |
| **ON** | **m** | **OM** | **f** | **-0.0833** | **-0.1326** | **-0.0341** | **<0.001** |
| OS | m | OM | f | -0.0646 | -0.1197 | -0.0095 | 0.0085 |
| OW | m | OM | f | -0.0464 | -0.1015 | 0.0087 | 0.1906 |
| **AO** | **m** | **ON** | **f** | **-0.1605** | **-0.2121** | **-0.1088** | **<0.001** |
| OC | m | ON | f | -0.0077 | -0.0570 | 0.0416 | 1.0000 |
| OE | m | ON | f | -0.0320 | -0.0837 | 0.0197 | 0.6604 |
| OM | m | ON | f | -0.0076 | -0.0593 | 0.0441 | 1.0000 |
| **ON** | **m** | **ON** | **f** | **-0.0752** | **-0.1245** | **-0.0259** | **<0.0011** |
| OS | m | ON | f | -0.0565 | -0.1116 | -0.0014 | 0.0396 |
| OW | m | ON | f | -0.0382 | -0.0933 | 0.0169 | 0.4795 |
| **AO** | **m** | **OS** | **f** | **-0.3717** | **-0.4234** | **-0.3200** | **<0.001** |
| **OC** | **m** | **OS** | **f** | **-0.2189** | **-0.2682** | **-0.1696** | **<0.001** |
| **OE** | **m** | **OS** | **f** | **-0.2432** | **-0.2949** | **-0.1915** | **<0.001** |
| **OM** | **m** | **OS** | **f** | **-0.2188** | **-0.2705** | **-0.1671** | **<0.001** |
| **ON** | **m** | **OS** | **f** | **-0.2864** | **-0.3357** | **-0.2371** | **<0.001** |
| **OS** | **m** | **OS** | **f** | **-0.2677** | **-0.3228** | **-0.2126** | **<0.001** |
| **OW** | **m** | **OS** | **f** | **-0.2494** | **-0.3045** | **-0.1943** | **<0.001** |
| **Comparison** | | | | **Results Tukey-HSD** | | | |
| species | sex | species | sex | diff | lwr | upr | p adjusted |
| **AO** | **m** | **OW** | **f** | **-0.1511** | **-0.2135** | **-0.0888** | **<0.001** |
| OC | m | OW | f | 0.0016 | -0.0587 | 0.0620 | 1.0000 |
| OE | m | OW | f | -0.0226 | -0.0850 | 0.0397 | 0.9910 |
| OM | m | OW | f | 0.0018 | -0.0606 | 0.0641 | 1.0000 |
| **ON** | **m** | **OW** | **f** | **-0.0658** | **-0.1262** | **-0.0055** | **0.0204** |
| OS | m | OW | f | -0.0471 | -0.1123 | 0.0181 | 0.4109 |
| OW | m | OW | f | -0.0289 | -0.0941 | 0.0363 | 0.9534 |

Significant comparisons are displayed in bold. The p value is adjusted for multiple comparisons. OW = *Oryzias wolasi*, OM = *O. matanensis*, OC = *O. celebensis*, ON = *O. nigrimas*, OE = *O. eversi*, OS = *O. sarasinorum*, AO = *Adrianichthys oophorus*, f = female, m = male.

**Table S5 Mean pelvic fin thickness metrics for the lateral and medial fin ray of female and male ricefishes.**

|  | **Females, lateral fin ray** | | | **Females, medial fin ray** | | |
| --- | --- | --- | --- | --- | --- | --- |
| **Specimen** | **CSA (mm²)** | **Diameter (µm)** | **Diameter/FL** | **CSA (mm²)** | **Diameter (µm)** | **Diameter/FL** |
| *A. oophorus* | 85824 | 330,57 | 27,97 | 72000 | 302,78 | 25,62 |
| *O. eversi* | 34896 | 210,79 | 32,62 | 57384 | 270,30 | 41,83 |
| *O. sarasinorum* | 43560 | 235,50 | 24,60 | 51984 | 257,27 | 26,87 |
| *O. nigrimas* | 14952 | 137,98 | 25,85 | 19824 | 158,87 | 29,76 |
| *O. celebensis* | 16128 | 143,30 | 38,73 | 15624 | 141,04 | 38,12 |
| *O. matanensis* | 23184 | 171,81 | 34,50 | 22104 | 167,76 | 33,69 |
| *O. wolasi* | 10584 | 116,09 | 34,09 | 9792 | 111,66 | 32,79 |
|  | **Males, lateral fin ray** | | | **Males, medial fin ray** | | |
| **Specimen** | **CSA (mm²)** | **Diameter (µm)** | **Diameter/FL** | **CSA (mm²)** | **Diameter (µm)** | **Diameter/FL** |
| *A. oophorus* | 23472 | 172,87 | 34,44 | 29088 | 192,45 | 38,34 |
| *O. eversi* | 26280 | 182,92 | 43,30 | 30600 | 197,39 | 46,72 |
| *O. sarasinorum* | 27072 | 185,66 | 36,40 | 23472 | 172,87 | 33,90 |
| *O. nigrimas* | 21672 | 166,11 | 33,22 | 23760 | 173,93 | 34,79 |
| *O. celebensis* | 16344 | 144,26 | 34,72 | 15696 | 141,37 | 34,02 |
| *O. matanensis* | 28080 | 189,08 | 36,75 | 27504 | 187,13 | 36,37 |
| *O. wolasi* | 11520 | 121,11 | 35,94 | 12456 | 125,93 | 37,37 |

Means have been calculated from the left and right pelvic fin. Cross section area (CSA) is the measure for pelvic fin thickness at both measurement points (lateral and medial fin ray). The diameter (D) of CSA was divided by fin length (FL) to account for the effect of fin size on fin ray thickness. When corrected for fin length, the CSA is not disproportionately increased in female pelvic brooders, but even larger in males.

**Table S6 Linear mixed effect model summary for pelvic fin ray thickness.**

| **Means of the lateral fin rays** | | | |  |  |  |  |
| --- | --- | --- | --- | --- | --- | --- | --- |
| **Fixed effect** |  |  | *numDF* | *denDF* | *F* | *β* | *P* |
| Standard length | |  | 1 | 10.05 | 0.3354 | 0.1418 | 0.5752 |
| Sex |  |  | 1 | 7.5621 | 5.7438 | 0.2519 | **0.0451** |
| Reproductive strategy | | | 1 | 9.7892 | 9.7892 | 0.471 | 0.098 |
| Fin length | | | 1 | 11.0258 | 84.4193 | 0.7234 | **<0.0001** |
| Sex*Reproductive strategy | | | 1 | 7.4064 | 0.0534 | -0.1262 | 0.8235 |
| **Random effect** | |  |  |  |  |  |  |
| Species |  |  | 1 |  |  |  | 0.0918 |
| **Means of the medial fin rays** | | | |  |  |  |  |
| **Fixed effect** | |  | *numDF* | *denDF* | *F* | *β* | *P* |
| Standard length | |  | 1 | 13.8436 | 7.18 | 0.2293 | **0.018** |
| Sex |  |  | 1 | 7.1873 | 96.2037 | 0.2283 | **<0.0001** |
| Reproductive strategy | | | 1 | 8.7007 | 12.5592 | 1.7528 | **0.0066** |
| Fin length | | | 1 | 10.0545 | 0.5182 | 0.1125 | 0.49 |
| Sex*Reproductive strategy | | | 1 | 7.8146 | 166.439 | -1.35 | **<0.0001** |
| **Random effect** | |  |  |  |  |  |  |
| Species |  |  | 1 |  |  |  | **0.0003** |

Summary of the linear mixed effect models obtained with the lmerTest R package for pelvic fin thickness. Significance levels of p < 0.05 are highlighted in bold. Beta-coefficient reference for sex, reproductive strategy and fin is ‘male’, ‘pelvic brooding’ and ‘right pelvic fin’ respectively. Means have been calculated from the left and right pelvic fin.

**Table S7 Linear mixed effect model summary for body cavity volume.**

| **Total body volume** |  |  | | | |  |  |  |  |  |
| --- | --- | --- | --- | --- | --- | --- | --- | --- | --- | --- |
| **Fixed effect** |  |  | | | | *numDF* | *denDF* | *F* | *β* | *P* |
| Standard length | |  | | | | 1 | 7.1866 | 246.2647 | 2.02 | **<0.0001** |
| Sex |  |  | | | | 1 | 6.1452 | 71.5233 | -0.44 | **0.0001** |
| Reproductive strategy | | | | | | 1 | 8.177 | 4.6183 | -1.69 | 0.07 |
| Sex*Reproductive strategy | | | | | | 1 | 6.375 | 9.0118 | 0.26 | **0.0222** |
| **Random effect** | | | | |  |  |  |  |  |  |
| Species | | |  | |  | 1 |  |  |  | **0.0005** |
| **Pre pelvic girdle volume** | | |  | |  |  |  |  |  |  |
| **Fixed effect** | | |  | |  | *numDF* | *denDF* | *F* | *β* | *P* |
| Standard length | | | | |  | 1 | 7.5113 | 26.2048 | 1.08 | **0.0011** |
| Sex |  | | | |  | 1 | 5.3703 | 5.3692 | -0.29 | 0.0647 |
| Reproductive strategy | | | | | | 1 | 6.2769 | 3.5694 | -0.2 | 0.1056 |
| Sex*Reproductive strategy | | | | | | 1 | 4.1033 | 0.5481 | -0.23 | 0.5 |
| **Random effect** | | | |  | |  |  |  |  |  |
| Species | |  | |  | | 1 |  |  |  | 0.0617 |
| **Post pelvic girdle volume** | |  | |  | |  |  |  |  |  |
| **Fixed effect** | |  | |  | | *numDF* | *denDF* | *F* | *β* | *P* |
| Standard length | | | |  | | 1 | 13.3361 | 23.5939 | 0.8 | **0.0003** |
| Sex |  | | |  | | 1 | 5.5851 | 8.306 | -0.14 | **0.0304** |
| Reproductive strategy | | | | | | 1 | 7.8312 | 1.2172 | -0.79 | 0.3027 |
| Sex*Reproductive strategy | | | | | | 1 | 6.5722 | 23.2881 | 0.63 | **0.0023** |
| **Random effect** | |  | | | |  |  |  |  |  |
| Species |  |  | | | | 1 |  |  |  | **0.0002** |

**Table S8 Collection numbers of specimens included**

| **Collection number** | **Species** | **Sex** | **Treatment** |  |
| --- | --- | --- | --- | --- |
| MZB25201 | Adrianichthys oophorus | female | µ-CT, Calipers | |
| MZB25202 | Adrianichthys oophorus | female | µ-CT, Calipers | |
| MZB25203 | Adrianichthys oophorus | female | µ-CT, Calipers | |
| MZB25204 | Adrianichthys oophorus | female | Calipers | |
| MZB25205 | Adrianichthys oophorus | female | Calipers | |
| ZFMKICH121920 | Adrianichthys oophorus | female | Calipers | |
| ZFMKICH121921 | Adrianichthys oophorus | female | Calipers | |
| ZFMKICH121922 | Adrianichthys oophorus | female | Calipers | |
| ZFMKICH121923 | Adrianichthys oophorus | female | Calipers | |
| ZFMKICH121924 | Adrianichthys oophorus | female | Calipers | |
| MZB25206 | Adrianichthys oophorus | male | µ-CT, Calipers | |
| MZB25207 | Adrianichthys oophorus | male | Calipers | |
| MZB25208 | Adrianichthys oophorus | male | Calipers | |
| ZFMKICH121925 | Adrianichthys oophorus | male | Calipers | |
| ZFMKICH121926 | Adrianichthys oophorus | male | Calipers | |
| MZB25209 | Oryzias sarasinorum | female | µ-CT, Calipers | |
| MZB25210 | Oryzias sarasinorum | female | µ-CT, Calipers | |
| MZB25211 | Oryzias sarasinorum | female | µ-CT, Calipers | |
| ZFMKICH121927 | Oryzias sarasinorum | female | Calipers | |
| ZFMKICH121928 | Oryzias sarasinorum | female | Calipers | |
| ZFMKICH121929 | Oryzias sarasinorum | female | Calipers | |
| MZB25212 | Oryzias sarasinorum | male | µ-CT, Calipers | |
| MZB25213 | Oryzias sarasinorum | male | Calipers | |
| ZFMKICH121930 | Oryzias sarasinorum | male | Calipers | |
| ZFMKICH121931 | Oryzias sarasinorum | male | Calipers | |
| ZFMKICH121932 | Oryzias eversi | female | Calipers | |
| ZFMKICH121933 | Oryzias eversi | female | Calipers | |
| ZFMKICH121934 | Oryzias eversi | female | µ-CT, Calipers | |
| ZFMKICH121935 | Oryzias eversi | female | µ-CT, Calipers | |
| ZFMKICH121936 | Oryzias eversi | female | Calipers | |
| ZFMKICH121937 | Oryzias eversi | female | µ-CT, Calipers | |
| ZFMKICH121938 | Oryzias eversi | male | Calipers | |
| ZFMKICH121939 | Oryzias eversi | male | Calipers | |
| ZFMKICH121940 | Oryzias eversi | male | Calipers | |
| ZFMKICH121941 | Oryzias eversi | male | µ-CT, Calipers | |
| ZFMKICH121942 | Oryzias eversi | male | Calipers | |
| ZFMKICH121943 | Oryzias nigrimas | female | µ-CT, Calipers | |
| ZFMKICH121944 | Oryzias nigrimas | female | µ-CT, Calipers | |
| ZFMKICH121945 | Oryzias nigrimas | female | µ-CT, Calipers | |
| ZFMKICH121946 | Oryzias nigrimas | female | Calipers | |
| ZFMKICH121947 | Oryzias nigrimas | female | Calipers | |
| ZFMKICH121948 | Oryzias nigrimas | female | Calipers | |
| ZFMKICH121949 | Oryzias nigrimas | male | Calipers | |
| ZFMKICH121950 | Oryzias nigrimas | male | Calipers | |
| ZFMKICH121951 | Oryzias nigrimas | male | Calipers | |
| ZFMKICH121952 | Oryzias nigrimas | male | Calipers | |
| ZFMKICH121953 | Oryzias nigrimas | male | Calipers | |
| ZFMKICH121954 | Oryzias nigrimas | male | µ-CT, Calipers | |
| ZFMKICH121955 | Oryzias celebensis | female | µ-CT, Calipers | |
| ZFMKICH121956 | Oryzias celebensis | female | µ-CT, Calipers | |
| MZB25214 | Oryzias celebensis | female | Calipers | |
| MZB25215 | Oryzias celebensis | female | Calipers | |
| MZB25216 | Oryzias celebensis | female | Calipers | |
| ZFMKICH121957 | Oryzias celebensis | male | µ-CT, Calipers | |
| ZFMKICH121958 | Oryzias celebensis | male | Calipers | |
| ZFMKICH121959 | Oryzias celebensis | male | Calipers | |
| ZFMKICH121960 | Oryzias celebensis | male | Calipers | |
| MZB25217 | Oryzias celebensis | male | Calipers | |
| MZB25218 | Oryzias celebensis | male | Calipers | |
| ZFMKICH41828 | Oryzias matanensis | female | µ-CT, Calipers | |
| ZFMKICH41553 | Oryzias matanensis | female | µ-CT, Calipers | |
| ZFMKICH41831 | Oryzias matanensis | female | Calipers | |
| ZFMKICH41829 | Oryzias matanensis | female | Calipers | |
| ZFMKICH121961 | Oryzias matanensis | female | Calipers | |
| ZFMKICH41551 | Oryzias matanensis | female | Calipers | |
| ZFMKICH41833 | Oryzias matanensis | male | µ-CT, Calipers | |
| ZFMKICH41835 | Oryzias matanensis | male | Calipers | |
| ZFMKICH41836 | Oryzias matanensis | male | Calipers | |
| ZFMKICH41832 | Oryzias matanensis | male | Calipers | |
| ZFMKICH41834 | Oryzias matanensis | male | Calipers | |
| ZFMKICH121962 | Oryzias wolasi | female | µ-CT, Calipers | |
| ZFMKICH121963 | Oryzias wolasi | female | µ-CT, Calipers | |
| ZFMKICH121964 | Oryzias wolasi | female | µ-CT, Calipers | |
| ZFMKICH121965 | Oryzias wolasi | male | µ-CT, Calipers | |
| ZFMKICH121966 | Oryzias wolasi | male | Calipers | |
| ZFMKICH121967 | Oryzias wolasi | male | Calipers | |
| ZFMKICH121968 | Oryzias wolasi | male | Calipers | |

**Table S9 Micro-CT scan settings**

| Collection number | Species | Sex | Scanner | kV | µA | Dimensions (Px) | Pixel size (µm) | Rotation steps (Degree) | Frame Averaging | Random movement |
| --- | --- | --- | --- | --- | --- | --- | --- | --- | --- | --- |
| MZB25201 | *Adrianichthys oophorus* | female | Skyscan 1173 | 48 | 154 | 1120x1120 | 70.92 | 0.4 | 7 | 15 |
| MZB25202 | *Adrianichthys oophorus* | female | Skyscan 1173 | 33 | 162 | 2240x2240 | 35.46 | 0.25 | 7 | 15 |
| MZB25203 | *Adrianichthys oophorus* | female | Skyscan 1173 | 48 | 154 | 1120x1120 | 70.92 | 0.4 | 7 | 15 |
| MZB25206 | *Adrianichthys oophorus* | male | Skyscan 1173 | 38 | 192 | 2240x2240 | 31.92 | 0.2 | 7 | 20 |
| ZFMKICH121934 | *Oryzias eversi* | female | Skyscan 1173 | 50 | 160 | 1120x1120 | 39.72 | 0.4 | 7 | 15 |
| ZFMKICH121935 | *Oryzias eversi* | female | Skyscan 1173 | 50 | 160 | 1120x1120 | 39.72 | 0.4 | 7 | 15 |
| ZFMKICH121937 | *Oryzias eversi* | female | Skyscan 1173 | 50 | 160 | 1120x1120 | 39.72 | 0.4 | 7 | 15 |
| ZFMKICH121941 | *Oryzias eversi* | male | Skyscan 1173 | 36 | 176 | 2240x2240 | 24.12 | 0.3 | 8 | 20 |
| MZB25209 | *Oryzias sarasinorum* | female | Skyscan 1173 | 50 | 160 | 1120x1120 | 51.07 | 0.4 | 7 | 15 |
| MZB25210 | *Oryzias sarasinorum* | female | Skyscan 1173 | 50 | 160 | 1120x1120 | 51.07 | 0.4 | 7 | 15 |
| MZB25211 | *Oryzias sarasinorum* | female | Skyscan 1173 | 50 | 160 | 1120x1120 | 51.07 | 0.4 | 7 | 15 |
| MZB25212 | *Oryzias sarasinorum* | male | Skyscan 1173 | 39 | 196 | 2240x2240 | 29.08 | 0.2 | 7 | 15 |
| ZFMKICH121955 | *Oryzias celebensis* | female | Skyscan 1173 | 50 | 160 | 1120x1120 | 29.8 | 0.4 | 7 | 15 |
| ZFMKICH121956 | *Oryzias celebensis* | female | Skyscan 1173 | 50 | 160 | 1120x1120 | 29.8 | 0.4 | 7 | 15 |
| ZFMKICH121957 | *Oryzias celebensis* | male | Skyscan 1173 | 40 | 200 | 2240x2240 | 17.38 | 0.2 | 8 | 20 |
| ZFMKICH41828 | *Oryzias matanensis* | female | Skyscan 1173 | 50 | 154 | 1120x1120 | 46.1 | 0.4 | 7 | 15 |
| ZFMKICH41553 | *Oryzias matanensis* | female | Skyscan 1173 | 50 | 154 | 1120x1120 | 43.97 | 0.4 | 7 | 15 |
| ZFMKICH41833 | *Oryzias matanensis* | male | Skyscan 1173 | 40 | 200 | 2240x2240 | 26.95 | 0.2 | 8 | 20 |
| ZFMKICH121943 | *Oryzias nigrimas* | female | Skyscan 1173 | 54 | 140 | 2240x2240 | 24.82 | 0.2 | 5 | 10 |
| ZFMKICH121944 | *Oryzias nigrimas* | female | Skyscan 1173 | 50 | 160 | 1120x1120 | 48.23 | 0.4 | 7 | 15 |
| ZFMKICH121945 | *Oryzias nigrimas* | female | Skyscan 1173 | 50 | 160 | 1120x1120 | 48.23 | 0.4 | 7 | 15 |
| ZFMKICH121954 | *Oryzias nigrimas* | male | Skyscan 1173 | 36 | 170 | 2240x2240 | 24.82 | 0.3 | 8 | 20 |
| ZFMKICH121963 | *Oryzias wolasi* | female | Skyscan 1173 | 40 | 200 | 1120x1120 | 19.86 | 0.4 | 7 | 20 |
| ZFMKICH121965 | *Oryzias wolasi* | male | Skyscan 1173 | 40 | 180 | 2240x2240 | 17.02 | 0.4 | 8 | 20 |
| ZFMKICH121962 | *Oryzias wolasi* | female | Skyscan 1272 | 60 | 166 | 1344x2016 | 12 | 0.2 | 5 | 15 |
| ZFMKICH121964 | *Oryzias wolasi* | female | Skyscan 1272 | 60 | 166 | 1344x2016 | 12 | 0.2 | 5 | 15 |
| MZB25202 | *Adrianichthys oophorus* | female | Skyscan 1272 | 56 | 156 | 1344x2016 | 12 | 0.1 | 5 | 15 |
| MZB25206 | *Adrianichthys oophorus* | male | Skyscan 1272 | 50 | 154 | 1344x2016 | 12 | 0.2 | 5 | 15 |
| ZFMKICH121932 | *Oryzias eversi* | female | Skyscan 1272 | 55 | 166 | 1344x2016 | 12 | 0.1 | 5 | 15 |
| ZFMKICH121933 | *Oryzias eversi* | female | Skyscan 1272 | 55 | 166 | 1344x2016 | 12 | 0.1 | 5 | 15 |
| ZFMKICH121936 | *Oryzias eversi* | female | Skyscan 1272 | 55 | 166 | 1344x2016 | 12 | 0.1 | 5 | 15 |
| ZFMKICH121941 | *Oryzias eversi* | male | Skyscan 1272 | 60 | 166 | 1344x2016 | 12 | 0.1 | 5 | 15 |
| MZB25210 | *Oryzias sarasinorum* | female | Skyscan 1272 | 50 | 160 | 1344x2016 | 12 | 0.2 | 5 | 15 |
| MZB25212 | *Oryzias sarasinorum* | male | Skyscan 1272 | 50 | 160 | 1344x2016 | 12 | 0.1 | 5 | 15 |
| ZFMKICH121955 | *Oryzias celebensis* | female | Skyscan 1272 | 51 | 160 | 1344x2016 | 12 | 0.1 | 5 | 15 |
| ZFMKICH121957 | *Oryzias celebensis* | male | Skyscan 1272 | 52 | 160 | 1344x2016 | 12 | 0.2 | 5 | 15 |
| ZFMKICH41553 | *Oryzias matanensis* | female | Skyscan 1272 | 50 | 160 | 1344x2016 | 12 | 0.1 | 5 | 15 |
| ZFMKICH41833 | *Oryzias matanensis* | male | Skyscan 1272 | 52 | 156 | 1344x2016 | 12 | 0.1 | 5 | 15 |
| ZFMKICH121943 | *Oryzias nigrimas* | female | Skyscan 1272 | 56 | 166 | 1344x2016 | 12 | 0.1 | 5 | 15 |
| ZFMKICH121944 | *Oryzias nigrimas* | female | Skyscan 1272 | 56 | 166 | 1344x2016 | 12 | 0.1 | 5 | 15 |
| ZFMKICH121945 | *Oryzias nigrimas* | female | Skyscan 1272 | 56 | 166 | 1344x2016 | 12 | 0.1 | 5 | 15 |
| ZFMKICH121952 | *Oryzias nigrimas* | male | Skyscan 1272 | 50 | 160 | 1344x2016 | 12 | 0.1 | 6 | 15 |
