# Supplementary material for: Complex sexually dimorphic traits shape the parallel evolution of a novel reproductive strategy in Sulawesi ricefishes (Adrianichthyidae)
Source: BMC Ecol Evol. 2021 Apr 20;21:57. doi: 10.1186/s12862-021-01791-z (PMC8056572; doi:10.1186/s12862-021-01791-z)
Supplement: Supplementary file 2 — Additional file 2. Additional figures. [file 12862_2021_1791_MOESM2_ESM.docx]

**Additional file 2 for: Complex sexually dimorphic traits shape the parallel evolution of a novel reproductive strategy in Sulawesi ricefishes (Adrianichthyidae).**

Tobias Spanke^1^, Leon Hilgers^1^, Benjamin Wipfler^1^, Jana M. Flury^1^, Arne W. Nolte², Ilham V. Utama³, Bernhard Misof^1^, Fabian Herder^1^ & Julia Schwarzer^1*^

^1^ Zoologisches Forschungsmuseum Alexander Koenig, Adenauerallee 160, 53113 Bonn, Germany

^2^ Carl von Ossietzky Universität Oldenburg, AG Ökologische Genomik, Carl von Ossietzky-Str. 9-11 26111 Oldenburg

^3^ Ichthyology Laboratory, Indonesian Institute of Sciences (LIPI), JL. Raya Jakarta-Bogor Km. 46, Cibinong 16911, Indonesia

^*^ Corresponding author: Julia Schwarzer

**Additional file 2: Supplementary Figures**

**
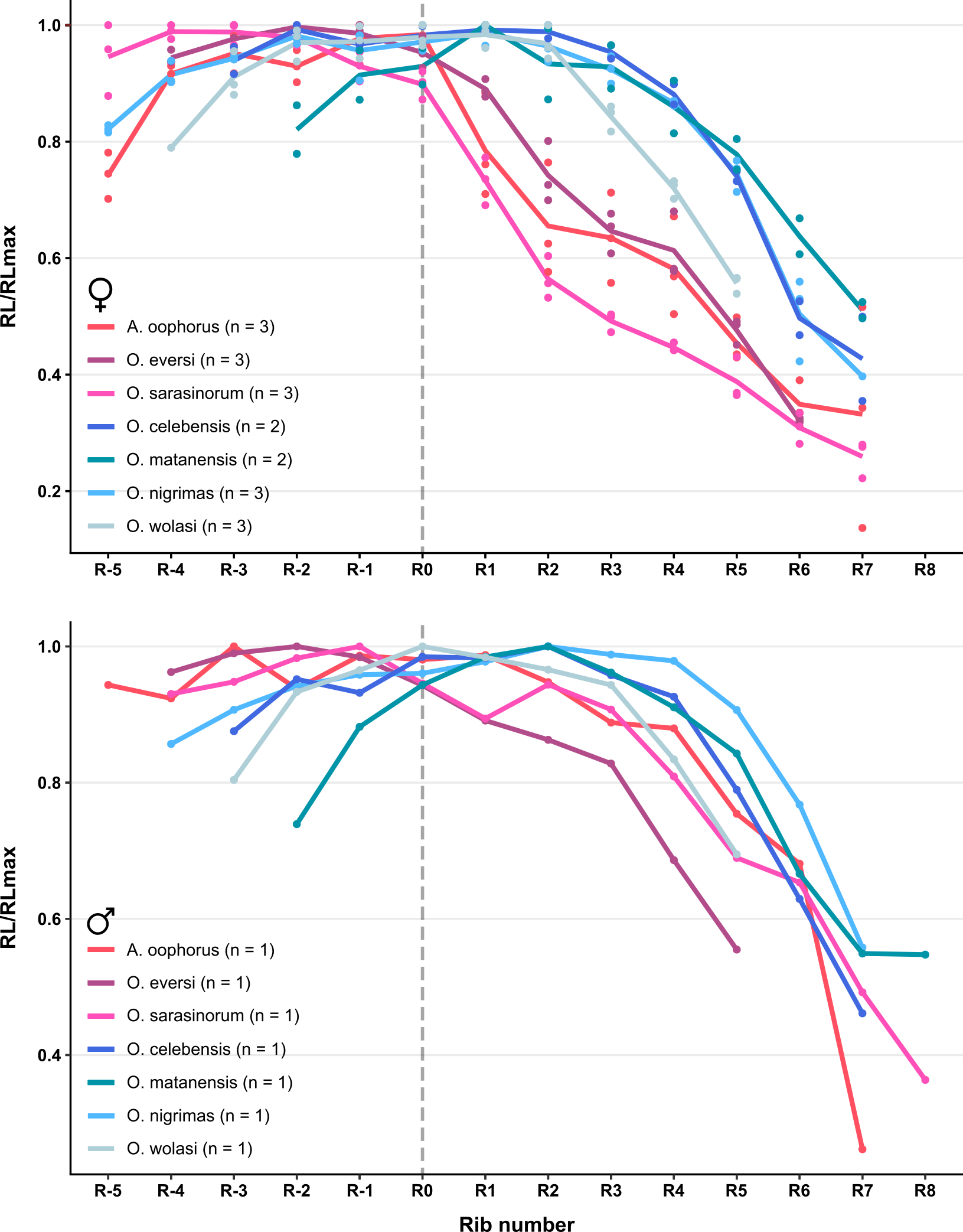
**

**Fig. S1** **Relative rib length of female and male ricefishes.** Relative rib length is calculated by dividing the length of each rib by the longest rib of the respective specimen. Dotted line (R0) marks the position of the lateral process and the insertion of the pelvic fin rays at the pelvic girdle. Top graph shows the reduction in rib lengths from R0 into the caudal direction of pelvic brooding females (red colors) in comparison to transfer brooding species (blue colors). Bottom graph shows this relation for male specimens of pelvic brooding and transfer brooding species.

**
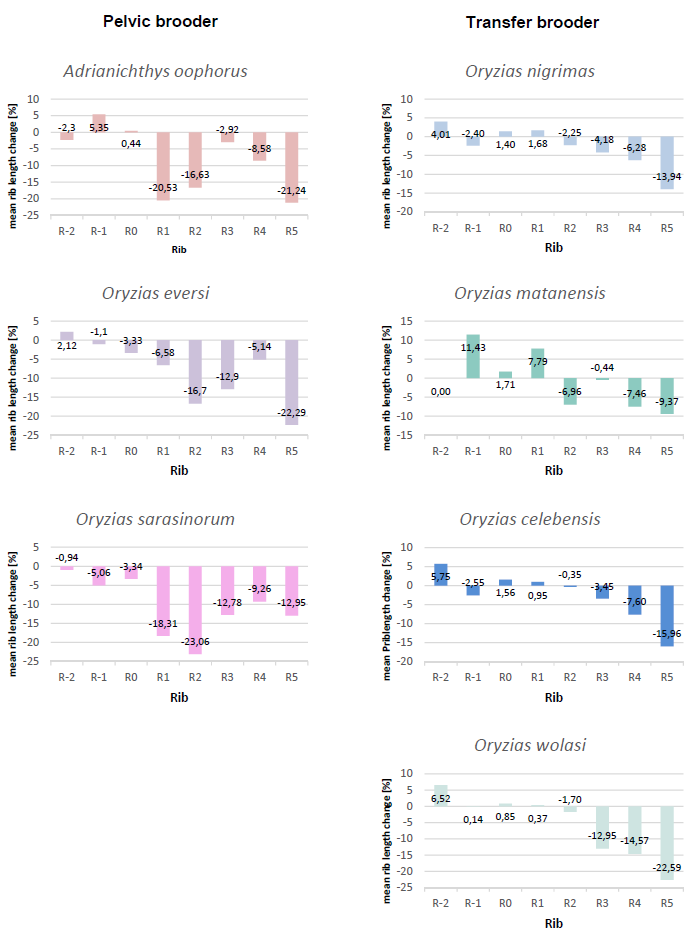
**

**Fig. S2** Bar charts showing mean gains and losses in rib length from one rib to the following for **female** pelvic (left row) and transfer (right row) brooding ricefishes. Negative numbers indicate a loss in rib length and positive numbers a gain in rib length.


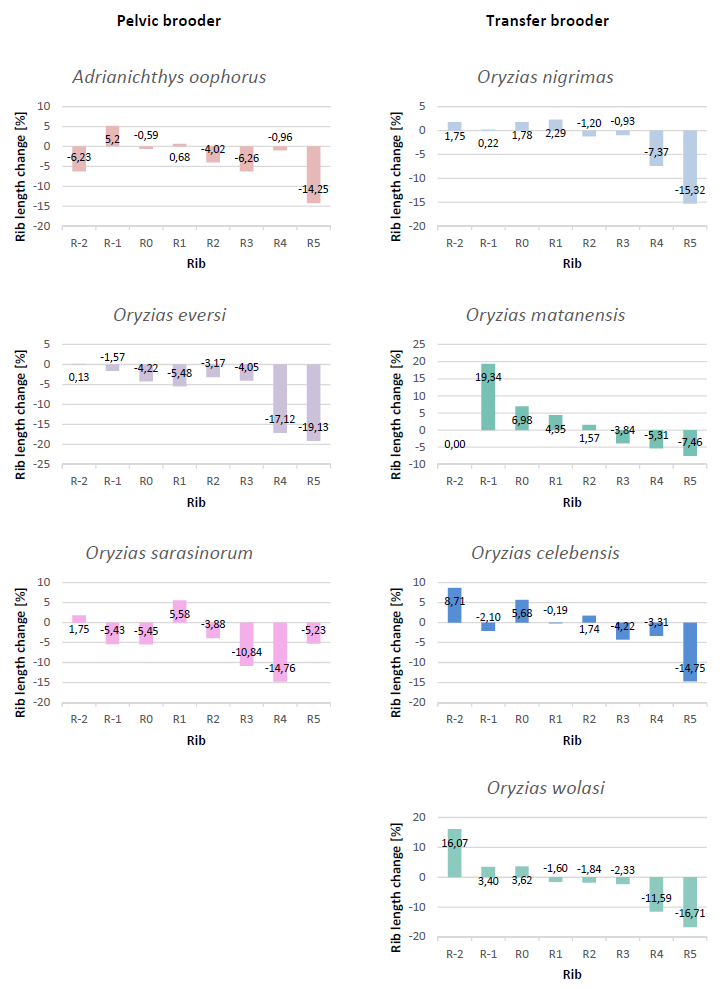


**Fig. S3** Bar charts showing mean gains and losses in rib length from one rib to the following for **male** pelvic (left) and transfer brooding (right) ricefishes. Negative numbers indicate a loss in rib length and positive numbers a gain in rib length.


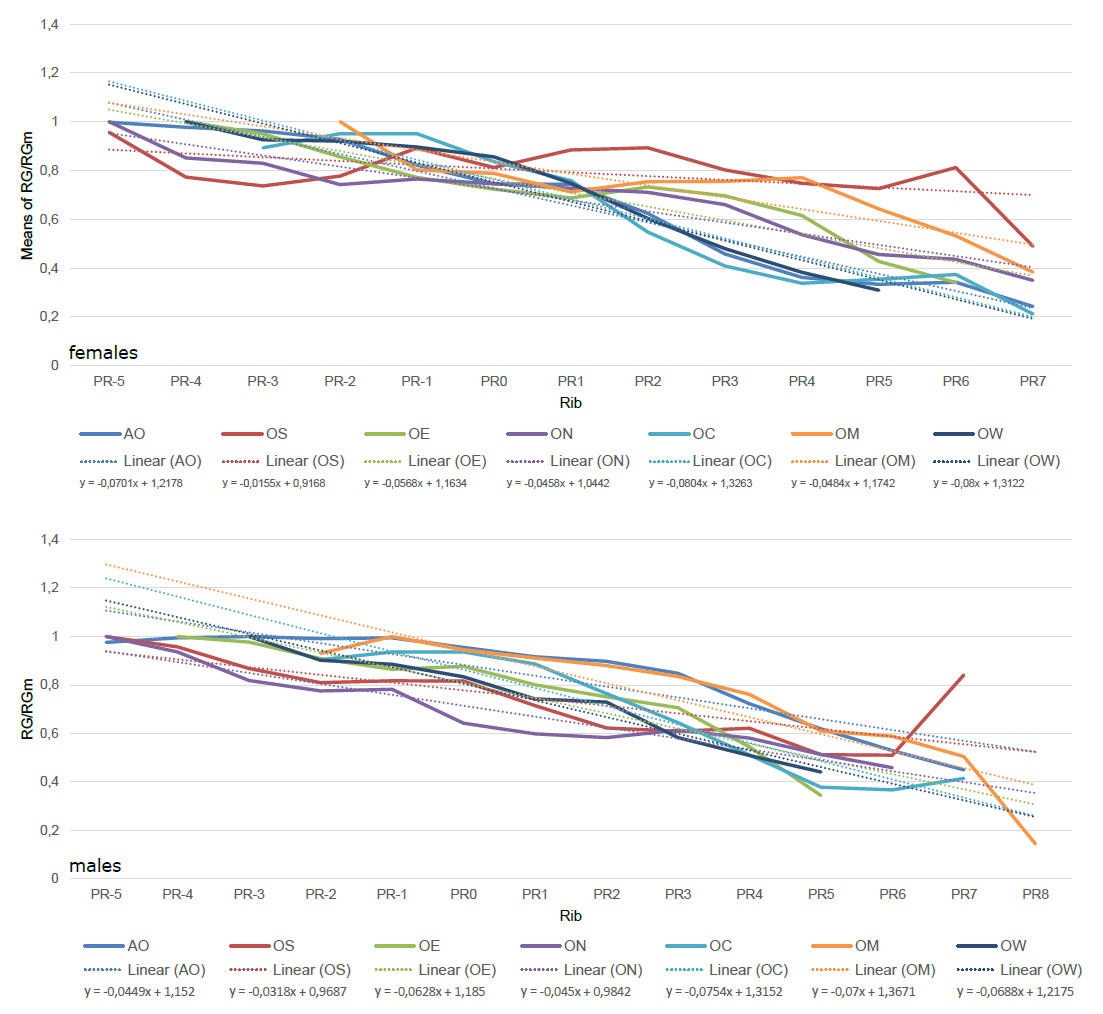


**Fig. S4** Line plots showing rib gap (i.e. the distance between the ventral tips of a rib pair) for female and male specimens. Generally, rib gap narrows down from anterior to posterior in female (top) and male (bottom) ricefishes. Linear mixed model analysis revealed reveal only a correlations of rib gap with reproductive strategy or sex for R-2, with transfer brooding males having slightly smaller gaps at this rib-pair. AO: *Adrianichthys oophorus*, OE: *Oryzias eversi*, OS: *O. sarasinorum*, ON: *O. nigrimas*, OM: *O. matanensis*, OW: *O. wolasi*.

**Fig. S5** Cross section area (CSA) of the lateral (top) and medial (bottom) pelvic fin rays in female (red) and male (blue) ricefishes. Mean values of the cross sectional areas of the left and right pelvic fin were taken.
